# Characterization of the deubiquitination activity and substrate specificity of the chicken ubiquitin-specific protease 1/USP associated factor 1 complex

**DOI:** 10.1371/journal.pone.0186535

**Published:** 2017-11-01

**Authors:** Hainan Zheng, Mengyun Wang, Chengcheng Zhao, Shanli Wu, Peifeng Yu, Yan Lü, Tiedong Wang, Yongxing Ai

**Affiliations:** 1 College of Animal Science, Jilin University, Changchun, China; 2 Institute of Translational Medicine, Jilin University, Changchun, China; 3 College of Basic Medical Sciences, Jilin University, Changchun, China; University of Florida, UNITED STATES

## Abstract

Deubiquitinases (DUBs) are essential regulators of intracellular processes involving ubiquitin (Ub) modification. The human DUB ubiquitin-specific protease 1 (hUSP1) interacts with human USP-associated factor 1 (hUAF1), and helps to regulate processes such as DNA damage repair. Previously, we identified a chicken USP1 homologue (chUSP1) during an investigation into the properties of Marek's disease virus (MDV). However, chUSP1's deubiquitination activity, interaction with chUAF1, and substrate specificity remained unknown. In the present study, we expressed and purified both chUAF1 and chUSP1 with or without putative catalytic core mutations using the Bac-to-Bac system, before investigating their deubiquitination activity and kinetics using various substrates. chUSP1 was shown to interact with chUAF1 both in cellular assays in which the two proteins were co-expressed, and in *in vitro* assays using purified proteins. Heterodimerization with chUAF1 increased the deubiquitination activity of chUSP1 up to 54-fold compared with chUSP1 alone. The chUSP1 mutants C91S, H603A, and D758A reduced the deubiquitination activity of the chUSP1/chUAF1 complex by 10-, 7-, and 33-fold, respectively, while the C91A and H594A chUSP1 mutants eliminated deubiquitination activity of the chUSP1/chUAF1 complex completely. This suggests that C91 and H594, but not D758, are essential for chUSP1 deubiquitination activity, and that a nucleophilic group at position 91 is needed for the deubiquitination reaction. The chUSP1/chUAF1 complex was found to have distinct substrate preferences; efficient hydrolysis of Ub dimers with K11-, K48-, and K63-linkages was seen, with weaker hydrolysis observed with K6-, K27-, and K33-linkages and no hydrolysis seen with a K29-linkage. Furthermore, other Ub-like substrates were disfavored by the complex. No activity was seen with SUMO1-GST, SUMO2- and SUMO3-dimers, ISG15-Rho, FAT10-Rho, or Ufm1-Rho, and only weak activity was observed with NEDD8-Rho. Overall, the data presented here characterize the activity and substrate preferences of chUSP1, and thus may facilitate future studies on its *in vivo* role.

## Introduction

The post-translational modification of proteins by ubiquitin (Ub), termed ubiquitination, dynamically modulates their stability and activity. During ubiquitination, isopeptide bonds are formed between the Ub carboxyl terminus and the ε-amino group of lysine (K) residues within the target protein, via sequential reactions catalyzed by E1 activating enzymes, E2 conjugating enzymes, and E3 ligases. Additionally, Ub possesses seven lysine residues (K6, K11, K27, K29, K33, K48, and K63) that can be ubiquitinated to form conjugated polyubiquitin chains of variable lengths and linkage types [1]. Modification of a protein by a K48-linked polyubiquitin chain generally targets the protein to the 26S proteasome for degradation [2], and while the cellular consequences of modification by K6-, K11-, K27-, K29-,or K33-linked polyubiquitin chains are not well understood, some studies have suggested that these modifications may also participate in the 26S proteasome-mediated degradation of target proteins [3]. Conversely, monoubiquitination and K63-linked polyubiquitination are widely known to regulate intracellular processes such as DNA damage repair, genomic stability, protein activity, inflammation, apoptosis, endocytic trafficking, and translation [2].

The deconjugation of Ub molecules from proteins is termed deubiquitination, and is performed by deubiquitinases (DUBs). Almost one hundred DUBs have been identified and classified in the human genome [4], with the largest subgroup, the ubiquitin-specific proteases (USPs), being known to participate in chromatin regulation [5, 6], virus infection [7–9], tumorigenicity [10], and immune regulation [11]. In particular, human USP1 (hUSP1) is critical for the regulation of DNA damage repair and genomic stability [12, 13]. Furthermore, by associating with human USP associated factor 1 (hUAF1), the deubiquitination activity of hUSP1 can be enhanced up to 35-fold [13]. The hUSP1/hUAF1 dimer has been shown to deubiquitinate monoubiquitinated FANCD2 and PCNA, thus modulating DNA damage repair and genomic stability [12, 13], and to regulate the degradation of T cell receptors and CD4+ molecules on the surface of T lymphocytes upon viral infection, leading to their functional impairment [14, 15].

Previously, we investigated the genomic integration preferences and pathogenic mechanisms of Marek's disease virus (MDV), a lethal avian herpesvirus capable of causing lymphomagenesis and immunosuppression in chickens, and discovered that chicken UAF1 as well as a putative USP-like protein might be involved in MDV pathogenesis (unpublished observation). Aligning this chicken USP-like protein with hUSP1 revealed that they shared 72% identity and a highly conserved catalytic core domain. While the existence of this USP-like protein was predicted previously in a study of chicken bursal lymphocytes [16], many questions remain regarding its function. In particular, it is unclear whether this USP-like protein deubiquitinates targets as hUSP1 does, and, if so, whether this activity is enhanced by chUAF1, and what types of Ub substrates are preferable for deconjugation.

In the current study, we aimed to investigate the properties of this novel chicken USP-like protein, which we termed chUSP1 because of its homology to hUSP1, by cloning the *chUAF1* and *chUSP1* genes. Having discovered that the wild type chUSP1 C-terminal mutations G680A and G681A abolished the autocleavage activity of wild type chUSP1, a chUSP1^GG680,681AA^ double mutant was used as a full-length control (chUSP1^FL^). Putative residues forming the catalytic core of chUSP1^FL^ were identified by homology analysis and mutated. Both full-length control and mutant proteins were expressed and purified using the Bac-to-Bac expression system. The substrate specificity and activity of either chUSP1^FL^ alone or the chUSP1^FL^ /chUAF1 complex was characterized using various Ub or Ub-like substrates. The resulting binding profiles could potentially be used in future investigations to determine the role of this complex in mechanisms related to viral infection, genomic integration, and immunosuppression in chickens.

## Materials and methods

### Construction of plasmids

The genes *chUSP1* (GenBank ID NM_001031290.1) and *chUAF1* (GenBank ID NM_ 001030964.1) were amplified from cDNA from chicken bursa of Fabricius tissue (Zyagen, San Diego, USA) by polymerase chain reaction (PCR) using the primers USP1 (F) and USP1 (R), and UAF1 (F) and UAF1 (R), respectively (Table 1). The *chUSP1* and *chUAF1* PCR products were either cloned into pFastBac-HTa to generate N-terminal hexa-His tagged chUSP1 or chUAF1 proteins, or cloned into pFastBac1 donor plasmids using NotI and XhoI restriction sites to generate untagged UAF1 protein using the Bac-to-Bac baculovirus expression system (Cat. No.10359-016, Invitrogen Corporation, Carlsbad, CA, USA), respectively. Mutations were introduced into the *chUSP1* gene using a QuikChange Site-Directed Mutagenesis kit (Cat. No.200524, Agilent Tech. Inc., Santa Clara, CA, USA.) according to the manufacturer’s instructions. The primers, USP1-GG (F) and USP1-GG (R), were used to generate the double mutant of the autocleavage site, wherein the GG residues at positions 680 and 681 are changed to AA. In this study, the chUSP1^GG680,681AA^ mutant was defined as the full-length control without other mutations, and was denoted chUSP1^FL^. The recombinant plasmid pFastBac-HTa-chUSP1^FL^ was used as a template to generate the putative catalytic core mutants using their respective primers listed in Table 1. TOP10 *Escherichia coli* competent cells were transformed with all recombinant pFastBac-HTa-chUSP1 and pFastBac1-chUAF1 plasmids, and then plated on agar Luria-Bertani (LB) medium containing 50 μg/ml ampicillin, and cultured at 37°C for 10 hours. Each recombinant plasmid was sequenced (Genewiz Inc., South Plainfield, NJ, USA) to confirm the correction of gene sequence and open reading frame before transformation of DH10Bac *Escherichia coli* competent cells for generation of recombinant bacmids.

**Table 1:** Primers for construction and mutation.

Primer name	Primer sequence
USP1 (F)	5’- CCGCCGCCCCCATGGGAATGCCGGGGGTGCTCCCGAG-3’
USP1 (R)	5’- CTGATGCAACTCGAGCAACAGGTGTATGCAAGTCCC-3’
UAF1 (F)	5’- CAGTGTCAAGCGGCCGCGAATGGCGGCGCATCACCGGCAG-3’
UAF1 (R)	5’- TACGGTCAACTCGAGGAGTAACCACGTGAGTCCAC-3’
C91S (F)	5’- GAATAATCTTGGCAACACCT**CG**TACCTTAACAGCGTTCTTCA-3’
C91S (R)	5’- TGAAGAACGCTGTTAAGGTA**CG**AGGTGTTGCCAAGATTATTC -3’
H594A (F)	5’- GGTTATTTGCAGTGGTGATG**GCA**AGTGGCATTACAATTAGCAG-3’
H594A (R)	5’- CTGCTAATTGTAATGCCACT**TGC**CATCACCACTGCAAATAACC-3’
H603A (F)	5’- GGCATTACAATTAGCAGCGGA**GCG**TACACAGCTTCTGTCAAAATC-3’
H603A (R)	5’- GATTTTGACAGAAGCTGTGTA**CGC**TCCGCTGCTAATTGTAATGCC-3’
D758A (F)	5’- CGAAGGGAAGTGGTTGCTTTTTG**CG**GATTCTGAAGTGAAAGTTAC-3’
D758A (R)	5’- GTAACTTTCACTTCAGAATC**CG**CAAAAAGCAACCACTTCCCTTCG-3’
USP1-GG (F)	5’- GGAAGCTGTGGGACTTCTTG**CA**G**C**ACAGAAGAGCAAGTCTGACTG-3’
USP1-GG (R)	5’- CAGTCAGACTTGCTCTTCTGT**G**C**TG**CAAGAAGTCCCACAGCTTCC-3’

The underlined sequence with straight line are the restriction sites NcoI in primer USP1 (F), XhoI in USP1 (R), NotI in UAF1 (F) or XhoI in UAF1 (R), respectively. The bolded sequence are the mutation sites.

### Expression and purification of chUSP1 and chUAF1 proteins

To generate recombinant expression bacmids, all recombinant pFast-chUSP1 and pFast-chUAF1 plasmids were used to transform DH10Bac competent cells. These cells were then cultured and plated onto selective agar containing 50 μg/ml kanamycin, 7 μg/ml gentamicin, 10 μg/ml tetracycline, 20 μg/ml 5-bromo-4-chloro-3-indolyl-β-d-galactopyranoside (X-gal) and 40 μg/ml isopropyl β-D-1-thiogalactopyranoside (IPTG), using the Bac-to-Bac expression system (Invitrogen) according to the manufacturer’s protocol. Transformed DH10Bac colonies were picked and replated onto selective medium three times to ensure that only transformed colonies were isolated. Recombinant bacmids were then extracted from the purified DH10Bac cells using an E.Z.N.A. Endo-Free BAC/PAC DNA kit (Cat. No. D2157-01,OMEGA Bio-tek Inc., Norcross, GA, USA) according to the manufacturer’s instructions. To generate recombinant baculovirus carrying the genes of interest, 2 ml of Sf9 cells were grown to 70% confluence in a 35 mm culture dish, then a mixture of 16 μl Cellfectin II transfection reagent (Invitrogen) and 2 μg recombinant bacmid was added. The transfected cells were then cultured in Sf-900 II SFM medium (Cat. No. 10902088, Invitrogen) for 7 days at 28°C and 5% CO_2_. The supernatant was harvested by centrifugation at 500 × *g* for 10 min at 4°C, 500 μl was added to Sf9 cells grown to 70% confluence in a 25 cm^2^ T-flask (Cat. No. 430639, Corning, NY, USA), and the cells were cultured for a further 72 hours to increase the recombinant baculovirus titer. The titer of the recombinant baculovirus-containing supernatant was determined using a FastPlax Titer Kit (Cat. No. 70850, EMD Millipore, Billerica, MA, USA), and after three generations the resulting baculovirus displayed a titer of more than 2 × 10^7^ pfu/ml. For the expression of recombinant proteins (chUSP1^FL^ and mutant chUSP1 or chUAF1, with or without a hexa-His tag), Sf9 cells were infected with recombinant baculovirus at a multiplicity of infection (MOI) of 1, grown for a further 72 hours, and then harvested. To express various chUSP1/chUAF1 complex, Sf9 cells were co-infected at a MOI of 1 with two individual recombinant baculoviruses carrying either hexa-His-tagged chUSP1 or untagged chUAF1, grown for 72 hours and then harvested. Harvested cells were washed in phosphate buffered saline before being lysed ultrasonically in lysis buffer (50 mM Tris HCl pH 8.0, 10% glycerol, 500 mM NaCl, and 10 mM imidazole). The lysate was clarified via centrifugation at 15,000 *× g* for 1 hour at 4°C followed by filtration through a 0.22-μm filter, and the supernatant was loaded onto a Ni-NTA agarose column (Cat. No. R90115, Thermo Fisher Scientific, Waltham, MA, USA). Following extensive washing with lysis buffer, bound proteins were eluted from the column in fractions using an elution buffer with an increasing imidazole gradient (50 mM Tris HCl pH 8.0, 10% glycerol, 500 mM NaCl, and 20–300 mM imidazole). The fractions containing recombinant protein were pooled and dialyzed against reaction buffer (50 mM HEPES-KOH pH 7.8, 20 mM NaCl, 0.1 mg/ml BSA, 0.5 mM EDTA, and 1 mM DTT). Purified protein aliquots were either used immediately or flash-frozen in liquid nitrogen and stored at -80°C.

### Analysis of the chUSP1-chUAF1 interaction

#### Analysis of the intracellular chUSP1-chUAF1 interaction using a co-expression assay

To investigate whether chUSP1 interacts with chUAF1 to form a complex within cells, Sf9 cells were co-infected at a MOI of 1 with two recombinant baculoviruses encoding either N-terminally hexa-His tagged chUSP1, with or without catalytic core mutations, or untagged chUAF1. Cells were harvested 72 hours after infection, and hexa-His tagged chUSP1 and any associated proteins were isolated on a Ni-NTA column as described above. Isolated proteins were then separated by sodium dodecyl sulfate-polyacrylamide gel electrophoresis (SDS-PAGE) using a 10% acrylamide gel, transferred onto a PVDF membrane, and examined by Western blotting using standard protocols. The presence of chUSP1 was determined using a mouse anti-hexa-His tag primary antibody at a dilution of 1:2000 (Cat. No. 66005-1-Ig, Proteintech, Rosemont, IL, USA) and an HRP-conjugated goat anti-mouse IgG secondary antibody at a dilution of 1:4000 (Cat. No.TA130003, OriGene, Rockville, MD, USA). The presence of chUAF1 was determined using an in-house rabbit anti-chUAF1 primary antibody at a dilution of 1:3000 and an HRP-conjugated goat anti-rabbit IgG secondary antibody at a dilution of 1:4000 (Cat. No.TA140003, OriGene). The in-house rabbit anti-chUAF1 polyclonal antibody was raised using recombinant GST-chUAF1 purified from BL21(DE3) *Escherichia coli* cells as an immunizing antigen. The hexa-His tagged chUAF1 purified from the Bac-to-Bac system, described above, was used as a coating antigen in an antiserum titration assay as follows; the antiserum was serially diluted and then titers were measured using an indirect enzyme-linked immunosorbent assay (ELISA) method according to standard protocols, using preimmunization serum as a negative control, and tetramethylbenzidine as the reaction substrate. The concentration of antibody in the serum was quantified by measuring the optical density (OD) at 450 nm (OD450), and the reciprocal value of the highest dilution, 64000:1, was defined as the titer of anti-chUAF1 serum at which the antiserum/control OD450 ratio was greater than 2.

#### Analysis of the *in vitro* chUSP1^FL^-chUAF1 interaction using a pull-down assay with purified proteins

To investigate the *in vitro* interaction between the chUSP1^FL^ and chUAF1 proteins, purified hexa-His tagged chUSP1^FL^ protein was used as bait in the following binding experiments. Excess hexa-His tagged chUSP1^FL^ was mixed with Ni-NTA resin to facilitate binding, and then unbound protein was removed with extensive washing with lysis buffer (see above). Sf9 cell lysate containing untagged chUAF1, the ‘prey’ protein, was then mixed with the chUSP1^FL^-bound resin in pre-cooled lysis buffer for 1 hour at 4°C to allow the chUSP1^FL^/chUAF1 complexes to form. The resin was washed extensively with lysis buffer to remove unbound proteins, and the protein complexes were eluted with elution buffer containing 300 mM imidazole (see above). The eluted proteins were identified by Western blotting using standard protocols, as described above.

#### Analysis of chUSP1 deubiquitination activity in the presence of chUAF1

Deubiquitination activity was assayed using fluorometry as follows. 100 nM Ub-7-amido-4-methylcoumarin (Ub-AMC, Cat. No.U-550, Boston Biochem, Cambridge, MA, USA) and 125 nM chUSP1^FL^ were mixed in a final volume of 100 μl 1× reaction buffer and incubated for 12 minutes at 37°C before the addition of 125 nM chUAF1. The mixture was incubated for a further 12 minutes at 37°C, and then the amount of AMC released from the Ub-AMC substrate was measured fluorometrically using a FluoroMax 4 fluorescence spectrophotometer (HORIBA Scientific, Edison, NJ, USA) with excitation and emission wavelengths of 380 nm and 460 nm, respectively. An assay mixture with equal volume of buffer lacking chUAF1 was used as a negative control.

### Deubiquitination assays

#### Investigating the preferred ubiquitin dimer link type

The substrate preference of chUSP1^FL^, either alone or in complex with chUAF1, was investigated by measuring the degree of deubiquitination of a variety of substrates. Reactions containing either 1 μM purified chUSP1^FL^ or 0.1 μM purified chUSP1^FL^ /chUAF1 complex and 98 μM of substrate (K6-, K11-, K27-, K29-, K33-, K48-, or K63-linked di-ubiquitin; Cat. No. UC-11B, UC-40B, UC-61B, UC-81B, UC-101B, UC-200, and UC-300B, respectively, Boston Biochem) in a final volume of 100 μl reaction buffer were carried out for 1 hour at 37°C. Reaction products were separated by SDS-PAGE and stained with Coomassie blue. The bands were quantified by densitometry using Image J software (National Institutes of Health, Bethesda, Maryland, USA) according to user guide [17]. Data represent the mean ± standard deviation, and are the average of at least three independent experiments.

#### Investigating deubiquitination substrate specificity

The substrate specificity of chUSP1^FL^ /chUAF1 complex-mediated deubiquitination was investigated using SUMO1-GST, di-SUMO2 or di-SUMO3 (Cat. No. UL-710, ULC-200, or ULC-300, respectively, Boston Biochem) and various rhodamine red-conjugated ubiquitin-like proteins as substrates. Assays measuring SUMO substrate preference were performed as described above, and reaction products were separated by SDS-PAGE and stained with Coomassie blue. Deubiquitination of rhodamine red-conjugated ubiquitin-like proteins was assessed as follows: hydrolysis reactions containing 0.1 to 1 μM of FAT10-rhodamine, NEDD8-rhodamine, UFM1-rhodamine, or ISG15-rhodamine substrates (Cat. No. UL-914, UL-835, UL-522, or UL-614, respectively, Boston Biochem), and 125 nM of chUSP1^FL^ /chUAF1 protein complex in a final volume of 100 μl 1× reaction buffer were allowed to proceed for 7 minutes at 37°C. The fluorescence intensity of the rhodamine dye that was released from the hydrolyzed substrates was then measured using a FluoroMax 4 fluorescence spectrophotometer (HORIBA Scientific) with excitation and emission wavelengths of 570 and 590 nm, respectively. Reactions containing each substrate with uncomplexed chUAF1 were used as negative controls. All experiments were performed in triplicate. The reaction velocity versus the substrate concentration was plotted in GraphPad Prism 7 software (GraphPad Software Inc., La Jolla, CA, USA) and curves were fitted described as below.

#### Investigating the kinetics of deubiquitination

The kinetics of deubiquitination of chUSP1^FL^ control and mutant variants of chUSP1 protein, alone or as part of the chUSP1/ chUAF1 complex, were assessed using Ub-AMC as a substrate. Deubiquitination reactions containing 0.1 to 1 μM Ub-AMC and proteins at the following concentrations were set up in 1× reaction buffer: 125 nM chUSP1^FL^, 20 nM chUSP1^FL^/chUAF1, 150 nM chUSP1^H603A^/chUAF1, 150 nM chUSP1^D758A^/chUAF1, 320 nM chUSP1^C91S^/chUAF1, 150 nM chUSP1^C91A^/chUAF1, 150 nM chUSP1^H594A^/chUAF1, or 150 nM chUSP1^CH91,594SA^/chUAF1. Preliminary experiments were performed to optimize the protein concentrations used in reactions and to ensure that their enzymatic activities were both comparable and within the detectable range. Enzymatic activity was assessed by measuring the fluorescence intensity of AMC released during the reaction using a FluoroMax 4 fluorescence spectrophotometer (HORIBA Scientific) with excitation and emission wavelengths of 380 nm and 460 nm, respectively, and enzyme kinetics were calculated as described below.

#### Analysis of the inhibition of deubiquitination activity

Inhibition of the deubiquitination activities of the chUSP1^FL^ control protein and mutant proteins was assessed using the irreversible DUB inhibitor ubiquitin vinyl sulfone (Ub-VS, Cat. No. U-212, Boston Biochem). 125 nM chUSP1^FL^ protein or chUSP1^FL^/chUAF1 protein complex and 4 μM Ub-VS were preincubated in 1×reaction buffer for 1 hour at 37°C, before the deubiquitination reaction was initiated by the addition of Ub-AMC to a final concentration of 100 nM. Enzymatic activity was assessed by measuring the fluorescence intensity of the AMC released during the reaction using a FluoroMax 4 fluorescence spectrophotometer (HORIBA Scientific) with excitation and emission wavelengths of 380 nm and 460 nm, respectively. Kinetics were calculated as described below.

#### Analysis of enzyme kinetics

The reaction velocities versus substrate concentrations for each experiment were plotted using GraphPad Prism 7 software (GraphPad Software Inc., La Jolla, CA, USA), and curves were fitted using the equation V = (Vmax• [S]) / ([S] + Km), which is Michaelis-Menten plot in GraphPad Prism 7. The kinetic constants *Km* and *V*_*max*_ were then determined using a Lineweaver-Burk plot, and the catalytic constant *k*_cat_ was defined as V_max_/enzyme concentration. The catalytic efficiency of the chUSP1^FL^ control and mutant DUBs were compared using the ratio *k*_*cat*_/*K*_*M*_.

## Results

### The chicken USP1 deubiquitinase shares a high level of homology with hUSP1

**Fig 1:**
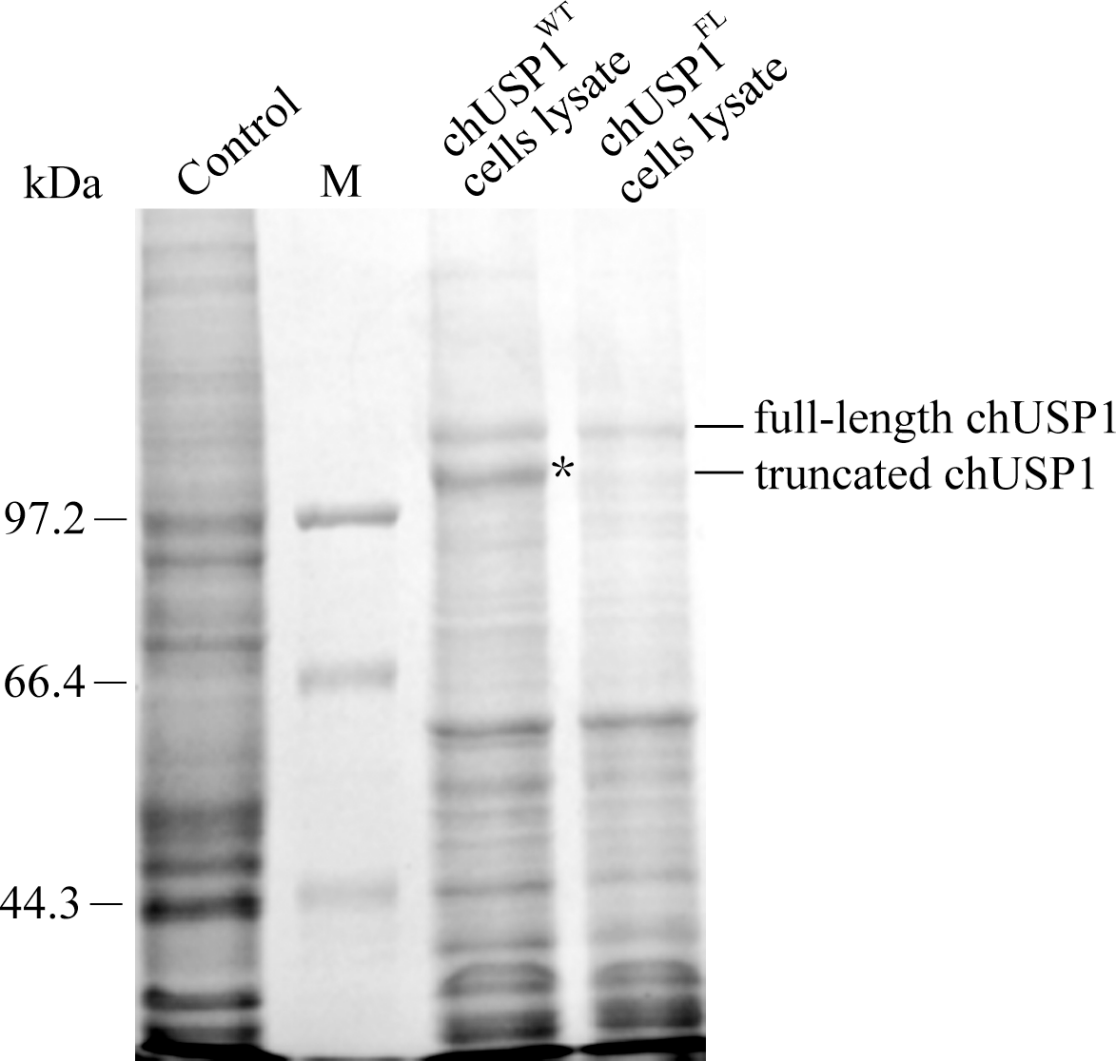
Expression of chUSP1 in insect Sf9 cells. Coomassie blue-stained SDS-PAGE image of Sf9 cells lysate, showing that mutation of the putative chUSP1 autocleavage residues G680A and G681A eliminated expression of the truncated form. Lane 1: Uninfected Sf9 control; lane 2(M): molecular markers (kDa); lane 3: chUSP1^WT^, S9 cells lysate expressing wild type chUSP1 before mutation of the G680 and G681 residues; lane 4: chUSP1^FL^, S9 cells lysate expressing chUSP1 with mutated residues (G680 and G681 residues to alanine) within the autocleavage site. Asterisk: The band of truncated chUSP1 presented in lane 3 but disappeared in lane 4.

In the human DUB USP1, a pair of glycine residues is known to be targeted for auto-cleavage and truncation. This autocleavage activity is also apparent in the wild-type chicken homologue chUSP1(chUSP1^WT^). As shown in Fig 1, lane 3, wild type chUSP1 is expressed in Sf9 cells as either a full-length or truncated protein, indicating the existence of the autocleavage site within the protein. The truncated version of this protein disappeared when two key residues (G680 and G681) within the autocleavage site were mutated to alanine (chUSP1^FL^, Fig 1, lane 4).

**Fig 2:**
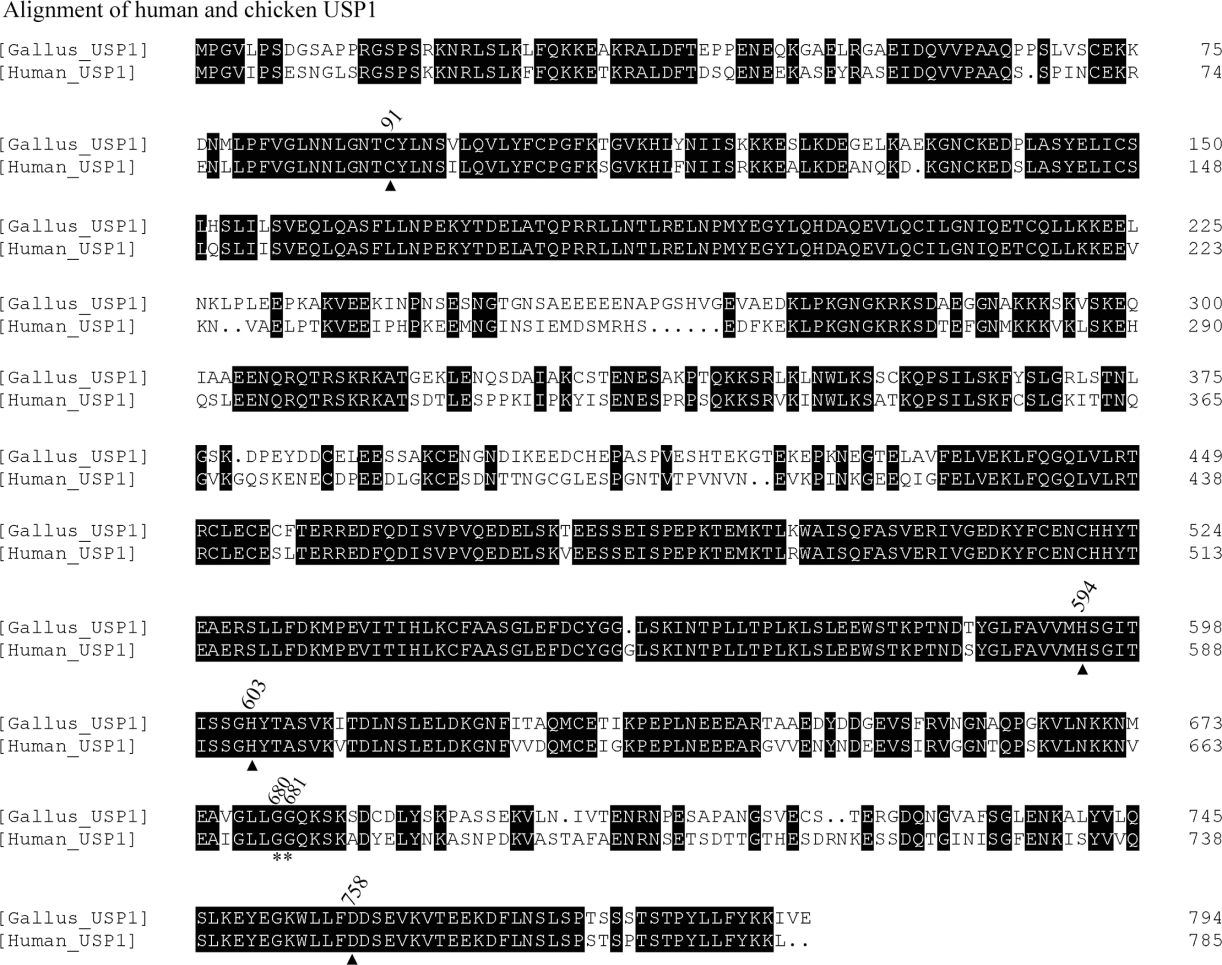
Alignment of chicken and human USP1 protein sequences. Alignment of chicken and human USP1 protein sequences showing approximately 72% identity. Considering the mutation sites in chUSP1, G680 and G681 are putative autocleavage sites (shown by asterisks) and C91, H594 (or, alternatively, H603) and D758 represent a putative catalytic triad (shown by triangles).

**Fig 3:**
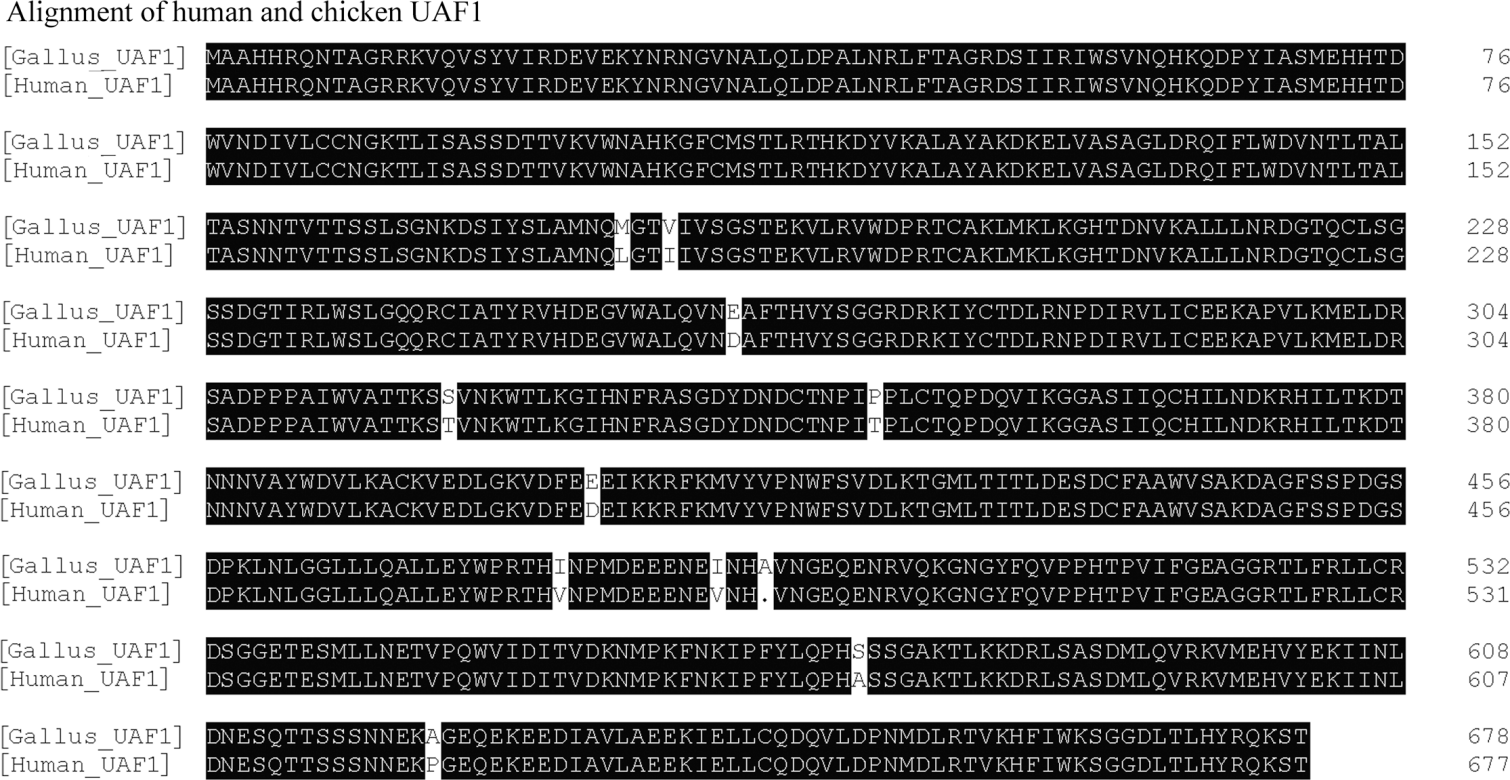
Alignment of chicken and human UAF1 protein sequences. Alignment of chicken and human UAF1 protein sequences showing 98% identity. Amino acid numbering is shown on the right of the figure.

Using DNAMAN 7 software (Lynnon LLC, San Ramon, CA, USA), aligning wild type of chUSP1 and hUSP1 revealed that the two proteins shared approximately 72% identity (Fig 2), and that the sequence flanking the autocatalytic GG motif (G670, G671 in hUSP1; G680, G681 in chUSP1; marked by asterisks in Fig 2) was highly conserved. Furthermore, the chicken and human UAF1 proteins shared approximately 98% identity (Fig 3), indicating a high level of conservation between the two species, and implying that the UAF1 proteins would behave similarly.

We hypothesized that G680 and G681 represent putative autocleavage sites in chUSP1, and generated a chUSP1^GG680,681AA^ double mutant, chUSP1^FL^, which we expressed in Sf9 cells. This protein was expressed in the full-length form but not the truncated form, confirming the location of the autocleavage motif in wild type chUSP1. As chUSP1^FL^ is expressed as only a single, full-length form, this protein was used as the baseline control in all subsequent activity assessment experiments.

We also identified highly conserved putative catalytic core residues in wild type chUSP1 based on the alignment, these being C91, H594 or H603, and D758 (Fig 2, marked by triangles). These residues were mutated to alanine or serine as described using chUSP1^FL^ gene as template, and the activity of the mutant enzymes was examined in subsequent experiments in order to characterize the putative catalytic core.

### chUSP1 and chUAF1 interact with each other

**Fig 4:**
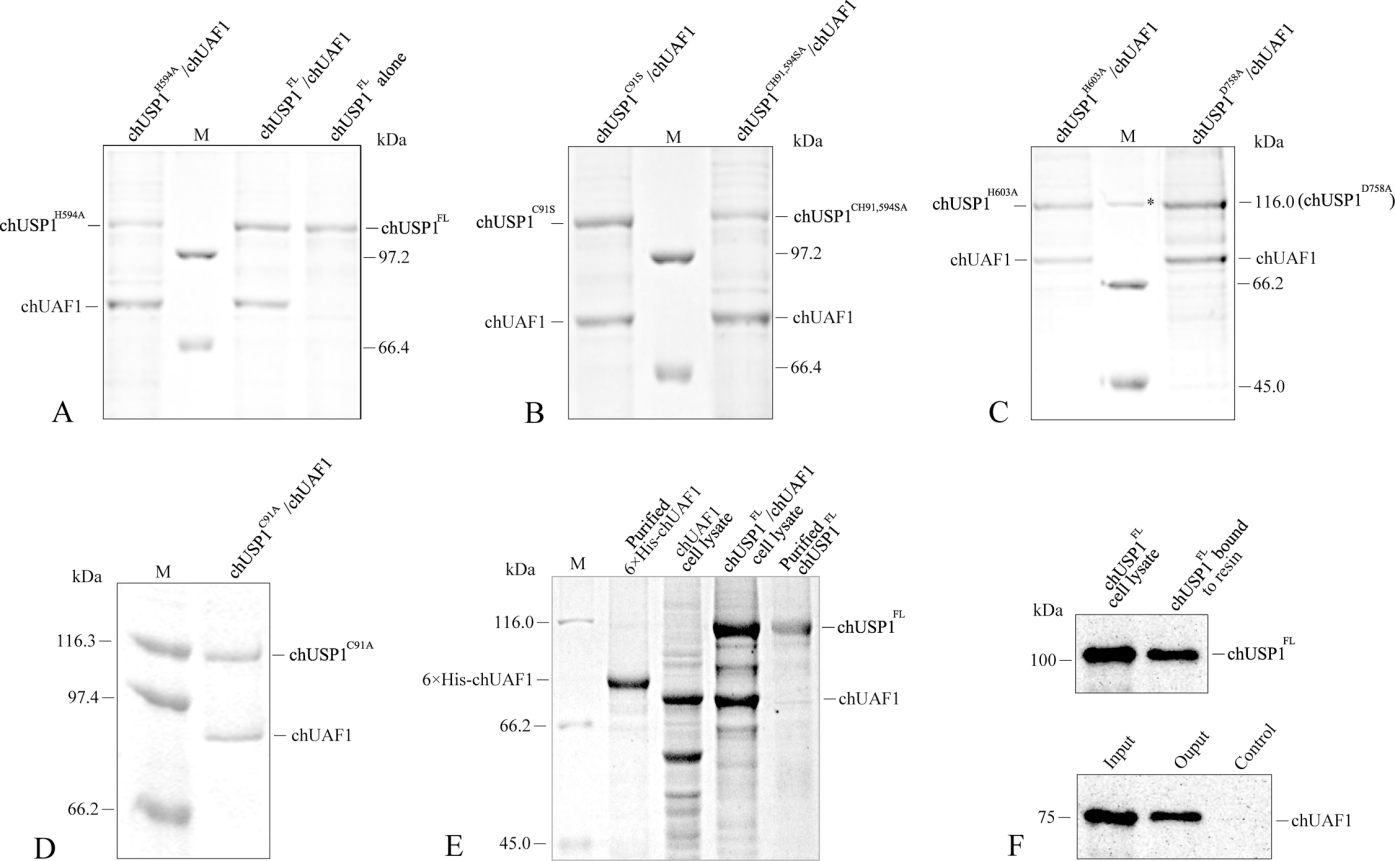
Interaction between chUSP1 and chUAF1. (A-D) Coomassie blue-stained polyacrylamide gels showing chUSP1 and chUAF1 proteins. Proteins were co-expressed in Sf9 cells where they formed intracellular complexes, and were then isolated using pull-down experiments with full-length or mutant chUSP1 as bait. (A) Lane 1, separated chUSP1^H594A^/chUAF1 complex proteins; lane 3, separated chUSP1^FL^/chUAF1 complex proteins; lane 4, chUSP1^FL^ alone. (B) Lane 1, separated chUSP1^C91S^/chUAF1 complex proteins; lane 3: separated chUSP1^CH91,594SA^/chUAF1 proteins. (C) Lane 1, separated chUSP1^H603A^/chUAF1 complex proteins; lane 3, separated chUSP1^D758A^/chUAF1 complex proteins. (D) Lane 2, separated chUSP1^C91A^/chUAF1 complex proteins. (E) Lane 2, purified 6×His tagged chUAF1 protein; lane 3, cell lysate expressing untagged chUAF1 protein; lane 4, cell lysate expressing chUSP1^FL^/chUAF1 complex proteins; lane 5, Purified chUSP1^FL^ protein. M: protein molecular markers (kDa); the top band labeled with asterisk is 116.0 kDa in (C). (F) Western blot confirming the presence of chUSP1 (using an anti-hexa-His tag antibody) and chUAF1 (using an anti-chUAF1 antibody), in both purified protein and cell lysates samples. Upper panel: Lane 1, cell lysate expressing chUSP1^FL^; lane 2, chUSP1^FL^ protein bound to NTA-Ni resin. Lower panel: Input, cell lysate expressing untagged chUAF1; Output, untagged chUAF1 eluted from chUSP1-bound Ni resin; Control, Ni resin without chUSP1^FL^ binding treated with untagged chUAF1 cell lysate, showing no chUAF1 binding.

Having shown that chicken USP1 and UAF1 displayed high levels of homology to their human counterparts, we investigated whether chUSP1 and chUAF1 were able to interact as the human forms do. Firstly, this interaction was investigated intracellularly by co-expressing untagged chUAF1 and hexa-His tagged chUSP1 with or without catalytic triad mutations within Sf9 cells. Subsequently, the *in vitro* interaction between the proteins was investigated with pulldown experiments using hexa-His tagged chUSP1^FL^ proteins as bait. We found that chUSP1, both in the absence of catalytic triad mutations and with the mutations C91S, C91S+H594A, H594A, H603A, or D758A, was capable of forming complexes with untagged chUAF1 that could be isolated (Fig 4A–4D and S1 Fig). This suggests that hexa-His tagged chUSP1 and untagged chUAF1 heterodimerized within Sf9 cells when co-expressed. Furthermore, as the chUSP1 mutants retained the ability to interact with chUAF1, the residues of the catalytic triad are not likely to impair the chUSP1-chUAF1 interaction. Similarly, pull-down assays with purified chUSP1^FL^ followed by Western blot analysis showed that untagged chUAF1 was bound to hexa-His tagged chUSP1^FL^ (Fig 4F), suggesting that the chUSP^FL^ /chUAF1 interaction could also occur in an extracellular context.

### chUAF1 augments chUSP1^FL^ activity

**Fig 5:**
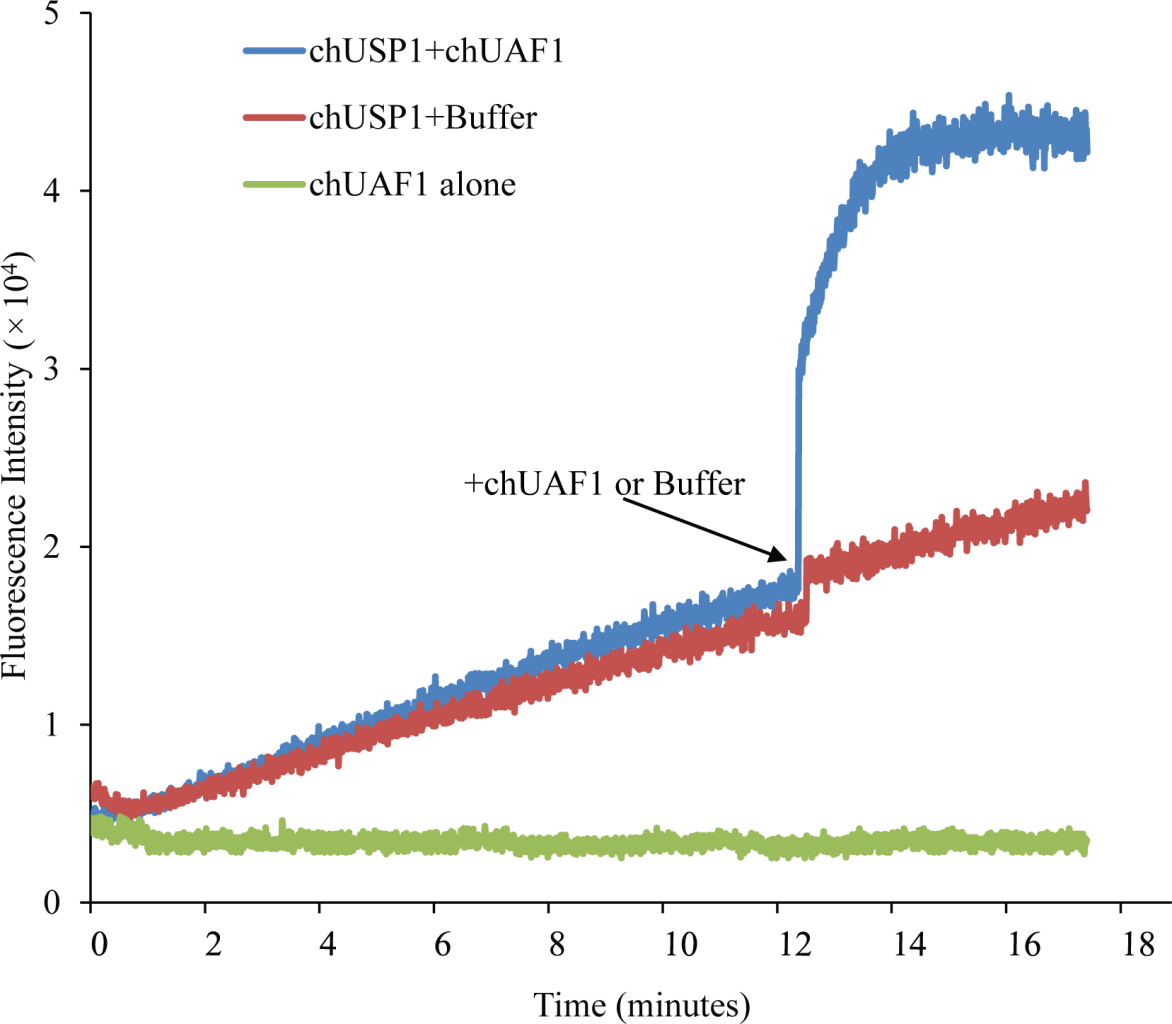
chUAF1 increases chUSP1^FL^ activity. Fluorescence intensity of AMC following the deubiquitination of Ub-AMC substrate by chUSP1^FL^. Deubiquitination with chUSP1^FL^ alone was allowed to proceed for 12 minutes before the addition of either chUAF1 (blue trace) or buffer (red trace). The reaction with chUAF1 alone (orange trace) in absence of chUSP1^FL^ was used as negative control.

As chUAF1 interacts with chUSP1^FL^, and the catalytic core mutants, we inspected whether chUAF1 could also increase chUSP1^FL^ activity by measuring the deubiquitination activity of chUSP1^FL^ before and after the addition of UAF1. As shown in Fig 5, three minutes after the addition of chUAF1, the deubiquitination activity of chUSP1^FL^ in an AMC fluorescence assay more than doubled as compared to the control containing chUSP1^FL^ but without chUAF1. As negative control, chUAF1 alone in absence of chUSP1^FL^ did not present deubiquitination activity.

### chUSP1^FL^ and chUSP1^FL^/chUAF1 deubiquitination substrate specificity

#### chUSP1^FL^/chUAF1 Ub hydrolysis activity is dependent on linkage type

**Fig 6:**
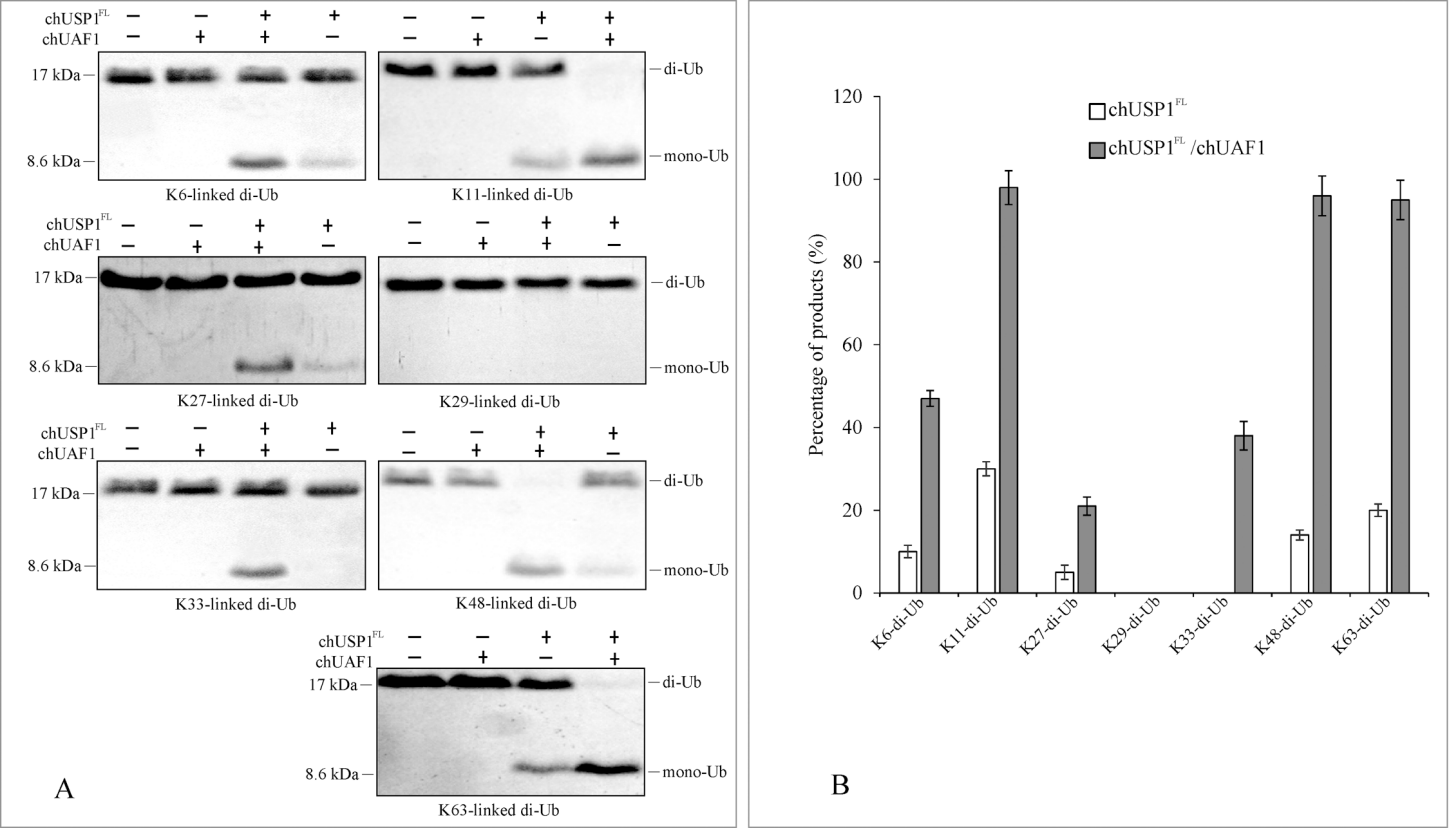
Analysis of chUSP1^FL^ and chUSP1^FL^ /chUAF1 hydrolysis activity with various substrate linkages. (A) Representative Coomassie blue-stained gel images showing the hydrolysis of di-Ub substrates with linkages at differing lysine (K) residues into mono-Ub substrates by chUSP1^FL^, chUAF1, or chUSP1^FL^/chUAF1 complex. The presence or absence of chUSP1^FL^ and chUAF1 is indicated above the lanes. The linkage type of the substrate is displayed below panels. (B) Bar chart showing the percentage of hydrolysis (intensity of mono-Ub substrate as a proportion of the lane total) for chUSP1^FL^ or chUSP1^FL^+chUAF1 with each substrate. The plotted data represent mean values ± standard deviation, and are the average of three independent experiments.

Since chUSP1^FL^ displays deubiquitination activity, which was enhanced further in the presence of chUAF1, we wanted to test the preferred ubiquitinated substrates for chUSP1^FL^ and chUSP1^FL^ /chUAF1. Firstly, we investigated the preferred Ub dimer linkage type for each molecular species using di-Ub substrates linked at various different lysine residues. chUAF1 alone demonstrated no isopeptidase activity with any of the tested substrates. chUSP1^FL^ alone could partly deubiquitinate K6-, K11-, K27-, K48- and K63-linked substrates, but not K29- and K33- linked substrates. Interestingly, the chUSP1^FL^/chUAF1 complex enhanced the observed levels of isopeptidase activity above those seen with chUSP1 alone on all substrates except K-29-linked substrate. A near complete deubiquitination activity is observed when K11-, K48-, and K63-linked substrates were used (Fig 6A and 6B). No isopeptidase activity was seen using the K29-linked substrate with either chUSP1^FL^ alone or the chUSP1^FL^ /chUAF1 complex. Overall, this data suggests that the hydrolysis activity of chUSP1^FL^ /chUAF1 is highly dependent on the substrate Ub chain linkage type.

#### chUSP1^FL^/chUAF1 specificity for Ub-like substrates

**Fig 7:**
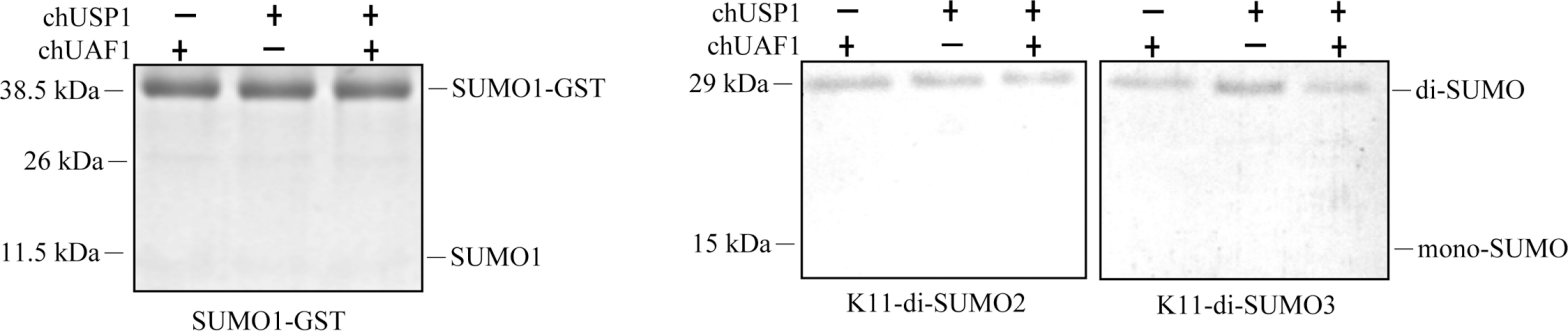
Hydrolysis of SUMO substrates by chUSP1^FL^ and chUSP1^FL^/chUAF1. Representative Coomassie blue-stained gel images showing the hydrolysis of either SUMO1-GST to SUMO1 (left panel) or K11-di-SUMO2 or K11-di-SUMO3 to mono-SUMO (right panel). The presence or absence of chUSP1^FL^ and chUAF1 is indicated above the lanes.

The ability of chUSP1^FL^ alone or with chUAF1, to hydrolyze a di-Ub substrate was dependent upon the type of K-linkage possessed by the substrate. We further investigated the hydrolysis activity of chUSP1^FL^/chUAF1 using a variety of ubiquitin-like substrates. Both chUSP1^FL^ and chUSP1^FL^/chUAF1 activity was tested against various SUMO substrates, but chUSP1^FL^, alone or in complex with chUAF1, was unable to hydrolyze SUMO1-GST, K11-linked di-SUMO2, or K11-linked di-SUMO3 (Fig 7).

**Fig 8:**
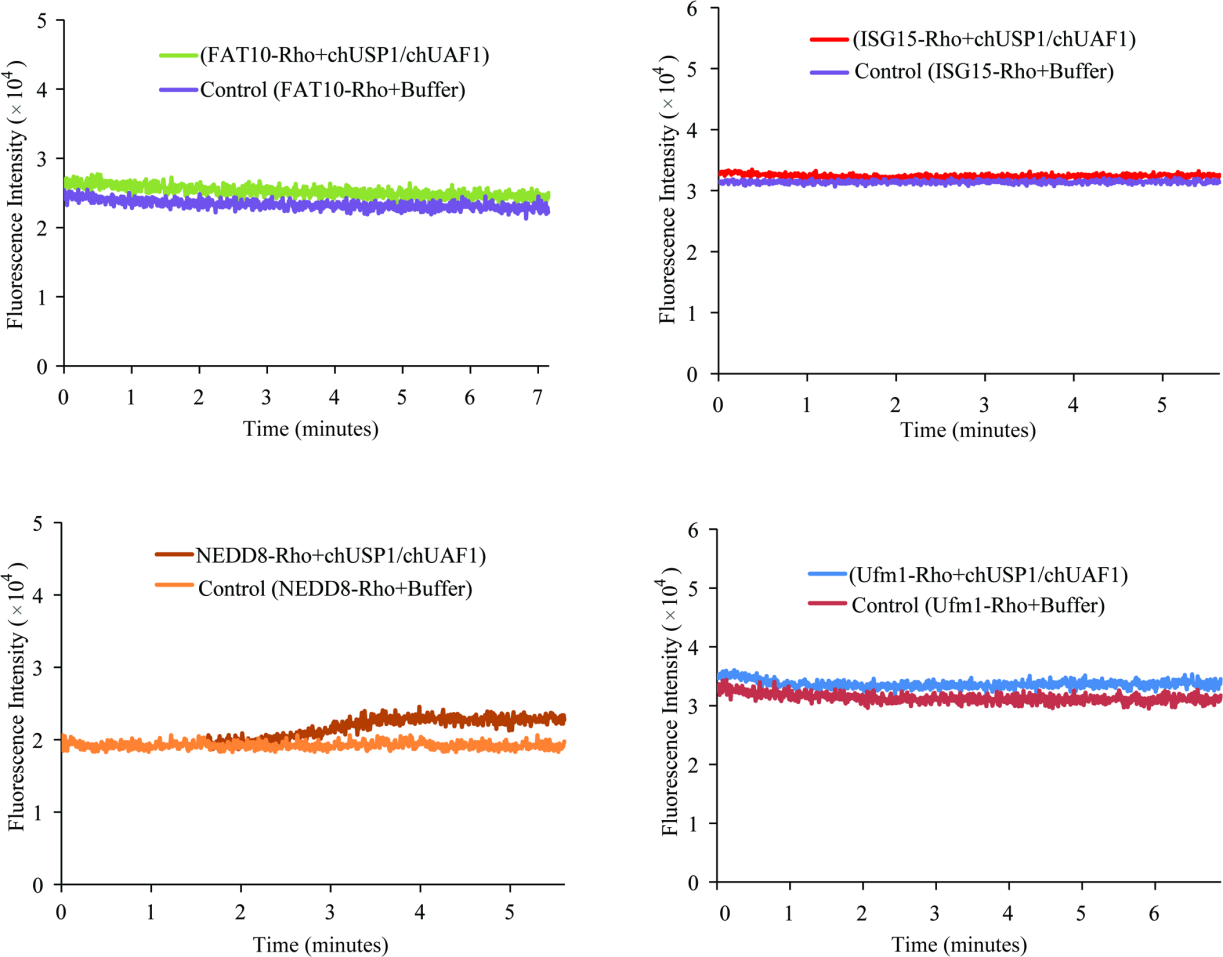
Hydrolysis of rhodamine-conjugated Ub-like substrates by chUSP1^FL^ /chUAF1. Fluorescence intensity of rhodamine released on the hydrolysis of the rhodamine-conjugated substrates FAT10 (A), ISG15 (B), NEDD8 (C), or Ufm1 (D). The fluorescence intensity traces of either chUSP1^FL^/chUAF1-containing reactions or negative controls containing an equal volume of buffer (Control) are shown for each substrate.

A similar trend was observed when the hydrolysis activity of the chUSP1^FL^/chUAF1 complex was investigated with other ubiquitin-like substrates conjugated to rhodamine dye. No significant hydrolysis was observed with the substrates FAT10, ISG15, and Ufm1, while some hydrolysis activity was seen with the NEDD8 substrate (Fig 8). Overall, this data suggests that chUSP1^FL^ displays no substrate cross-reactivity.

### Deubiquitination kinetics of chUSP1^FL^ catalytic core mutants

**Fig 9:**
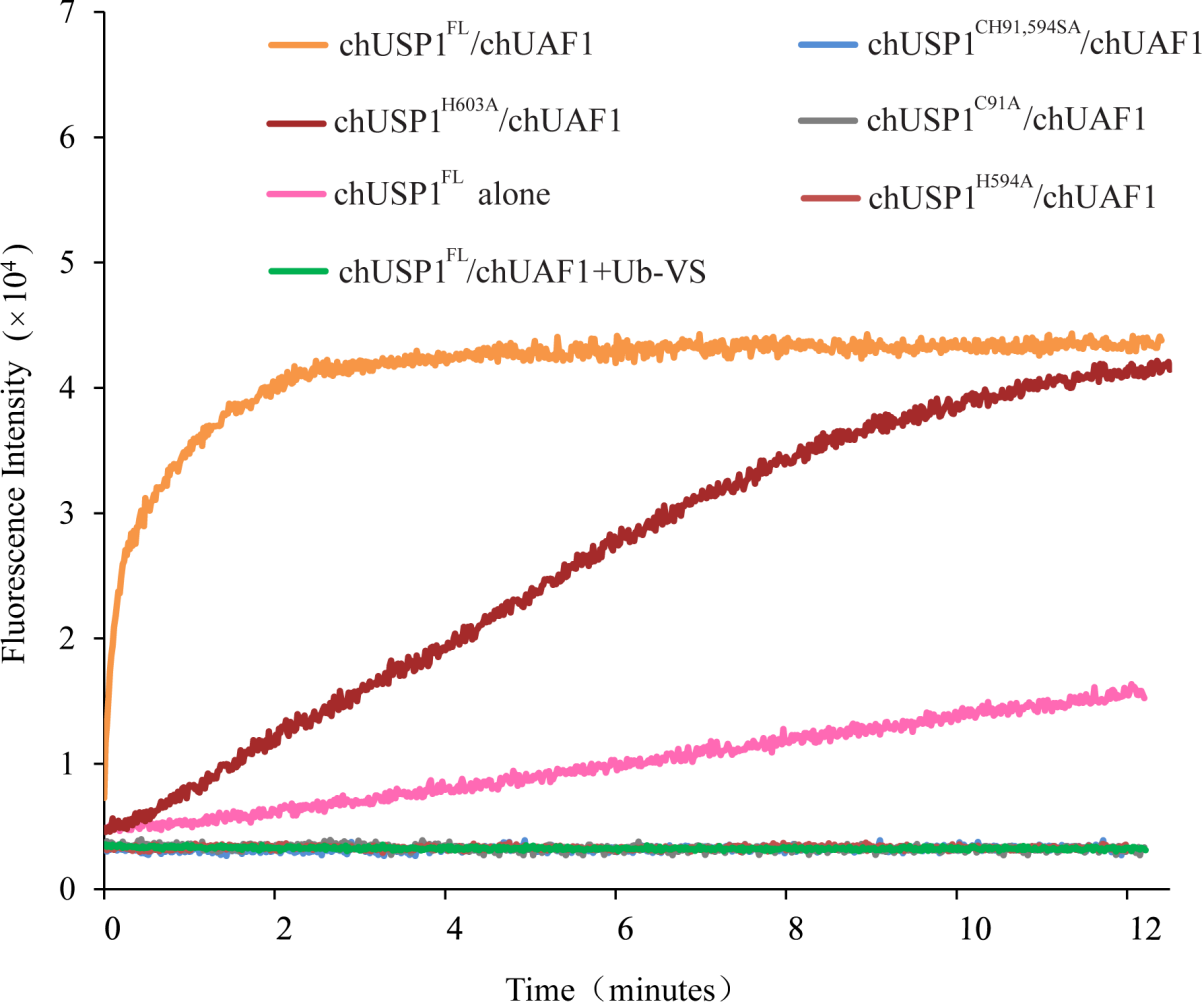
Activity of chUSP1^FL^ catalytic core mutants. Fluorescence intensity traces showing the release of AMC from Ub-AMC over time with chUSP1^C91A^ /chUAF1(gray), chUSP1^H594A^ /chUAF1(red), chUSP1^H603A^ /chUAF1(brown), and chUSP1^CH91,594SA^ /chUAF1(blue) protein complexes. chUSP1^FL^/chUAF1(orange) and chUSP1^FL^ alone(pink) were used as positive controls, For comparison, the activity of chUSP1^FL^/chUAF1 with the inhibitor Ub-VS(green) is also shown. chUSP1^FL^ catalytic core mutants were used at a concentration of 150 nM.

The deubiquitination activity of the putative chUSP1^FL^ catalytic triad mutants described previously was investigated relative to the non-cleavable full length enzyme by monitoring the hydrolysis of Ub-AMC substrate. Kinetic analysis showed that the chUSP1 mutants C91S, H603A, and D758A displayed lower levels of hydrolysis activity in the presence of chUAF1 than did chUSP1^FL^, shown by larger *K*_*M*_ and smaller *k*_cat_ values, but remained more active than chUSP1^FL^ alone (Table 2 and S2 Fig). The H603A mutant in complex with chUAF1 displayed a 3-fold increase in *K*_M_ and 7-fold decrease in catalytic activity (measured as *k*_cat_/*K*_M_) when compared to chUSP1^FL^ (Table 2 and S2 Fig). Similarly, the D758A mutant in complex with chUAF1 displayed an approximately 4-fold increase in *K*_M_ value and an approximately 33-fold decrease in catalytic activity. Interestingly, the C91S mutant in complex with chUAF1 showed a similar *K*_M_ to chUSP1^FL^, but approximately 10-fold lower catalytic activity. Overall, these data indicate that these catalytic core mutations did not completely abolish the deubiquitinating activity of chUSP1^FL^. Conversely, however, the mutations C91A and H594A did completely eliminate the deubiquitinating activity of chUSP1^FL^, and no catalytic activity was observed when protein was added up to a maximum concentration of 2 µM (data not shown). Additionally, these mutations reduced the deubiquitination activity of chUSP1^FL^ to a similar level as that observed with the inhibitor Ub-VS (Fig 9).

**Table 2:** Kinetic analysis of chUSP1 and chUSP1/chUAF1.

	*K*_M_ (μM)	*k*_cat_ (s^-1^)	*k*_cat_/*K*_M_ (mM^-1^s^-1^)
chUSP1^FL^ alone	2.471	0.0123±0.0011	4.9777
chUSP1^FL^/chUAF1	0.860	0.23139±0.009	269.0581
chUSP1^C91S^/chUAF1	1.0472	0.02949±0.00010	28.1608
chUSP1^H603A^/chUAF1	2.7999	0.105±0.0046	37.5013
chUSP1^D758A^/chUAF1	3.505	0.028494±0.0032	8.1295

Data are displayed as mean ± standard deviation, and are the average of three independent experiments.

## Discussion

In this study, we have identified and characterized a USP-like protein in chicken, chUSP1, which shows a high level of homology with its human counterpart. Similarly, as in humans, chicken UAF1 significantly augmented chUSP1 deubiquitinase activity, and this activity was found to be substrate dependent with distinct preferences for particular Ub linkages. The putative catalytic core residues C91, H594, H603, and D758 were confirmed to be important for chUSP1’s activity, with C91 and H594 being indispensable.

Protein ubiquitination is an important system of cellular regulation that is required for the maintenance of intracellular homeostasis. DUBs, of which there are approximately 100, are the primary enzymes that catalyze deubiquitination and are emerging targets for drug discovery [18], although their regulatory mechanisms remain poorly understood. USP1 is a member of the largest DUB superfamily, and is essential for DNA damage repair in human and in chicken cells [13, 19]. The action and expression of hUSP1 depend on the phase of cell cycle, and synchronize with other proteins related to DNA synthesis or damage repair, such as Rad51 and PCNA. hUSP1, either in mRNA level or in protein level, is cell-cycled regulated, and approach the maximum during S phase [20]. DNA damage suppresses its expression or induces its degradation [13]. Furthermore, hUSP1 has been shown to help to maintain stem cell characteristics in both osteosarcoma cells and mesenchymal stem cells by deubiquitinating and thus stabilizing inhibitors of DNA binding, which in turn antagonize the basic helix-loop-helix transcription factors responsible for differentiation [21].Both full-length and C-terminally truncated USP1 isoforms are able to deubiquitinate PCNA-Ub and FANCD2-Ub, and to regulate the DNA damage repair response and hence genomic stability [13, 22]. However, autocleavage of hUSP1 *in vivo* could result in down-regulation and subsequently degradation of hUSP1 activity. This is critical for regulating hUSP1 activity during DNA damage monitoring and repairing[22, 23]. Autocleavage sites mutated version of human USP1 were used to investigate its function *in vivo* or *in vitro*[18, 22, 24, 25]. This is why we used autocleavage sites mutant of chUSP1 to mimic native full-length protein for investigation of its deubiquitinating properties.

Studies have demonstrated that hUAF1, containing eight WD40 repeats, binds to hUSP1, hUSP12 and hUSP46, and enhances these DUBs activity[12, 26–28]. hUAF1 interacts with any of these three DUBs through its WD40 repeats, where the second repeat is essential for the interactions[12]. The contribution of hUSP1 to the interaction between hUSP1 and hUAF1 have been explored previously. Villamil et al. found that phosphorylation of Serine residues, S313, on hUSP1 promote the formation of the hUSP1/hUAF1 complex[29], although this Serine residue and its flanking sequence are not conserved between human and chicken. However, a conflictive point is that a non-phosphorylatable S313A mutation on hUSP1 did not impact the binding of hUSP1 to hUAF1, while the deletion of hUSP1 fragment 420–520 could demolish their interaction[30]. We discovered that, like their human counterparts, chUAF1 could bind to chUSP1, both when they were co-expressed and as purified proteins, and that this binding enhanced chUSP1 activity as much as 54-fold, much higher than the reported 35-fold enhancement in human USP1 full-length [12, 13, 31] and 16-fold in truncated human USP1 that missing N-terminal 20 amino acids (hUSP1ΔN) [24]. It was observed that *k*_cat_ of hUSP1 and hUSP1ΔN activity were increased respectively 18.6-fold[13] and 7-fold[24] in the presence of hUAF1, while the *k*_cat_ of chUSP1^FL^ was increased 21-fold in current study. *K*_M_ of chUSP1^FL^ decreased 2.87-fold in the presence of chUAF1, while *K*_M_ of hUSP1 decreased 1.5-fold [31]or 2-fold [12, 13]. These may suggested that the activation of USP1 upon binding to UAF1 may be due to an increase in *k*_cat_ with no drastic change in the *K*_M,_ that was similar to what were observed on activation of human USP12 and USP46 binding to hUAF1 [24]

Besides hUSP1, hUAF1 stimulates hUSP12 and hUSP46 activity as well. The crystallography analysis shows that the charged surface on hUAF1 β-propeller is the key for the interaction between hUAF1 and hUSP46 finger subdomain[26]. Their catalytic triad (Cys, His, Asp) locates in palm subdomain of three USPs. The hUAF1 stimulation on deubiquitinase activity of USP46 could be eliminated by hydrophobic mutation of residues involved in their interaction[26]. hUSP1/hUAF1 shares similar mode of interaction with USP46/UAF1 complex[26]. hUAF1 also can interact with finger domain of hUSP12 to activate hUSP12 [27]. These studies on structural analysis of hUSP12 and hUSP46, including interaction with hUAF1, may contribute to the understanding of the mechanism by which hUAF1 activates hUSP1. However, the activated complex of hUSP12 or hUSP46 by binding to hUAF1 could be activated further by binding to WDR20, another WD repeat protein, or binding to second hUAF1. This hyper-activation is not observed for the hUSP1/hUAF1 complex[28, 32]. In addition, hUSP1 (785 residues) is larger than hUSP12 and hUSP46 (370 and 366 residues, respectively). hUSP1 is 31% identical to hUSP12 and hUSP46, although the latter two proteins share 88% identity. The crystallography analysis of hUSP1/hUAF1 complex has not been done yet. Although chUAF1 shares 98% identity with hUAF1, the homology of USP1 is only 72% between the two species. Thus, further investigation is needed to explore how chUSP1 interacts with chUAF1, as compared to the interaction between hUSP1 and hUAF1.

Some proteases possess a catalytic triad, commonly Ser/His/Asp in serine proteases or Cys/His/Asp in cysteine proteases. This catalytic triad generates a nucleophile with which to attack the carbonyl group of a target peptide, and thus enables hydrolysis. The residue that is critical in this nucleophilic attack varies depending on the type of protease, with serine being essential in serine proteases and cysteine being essential in cysteine proteases. In order to generate a nucleophile, however, all that is required is the presence of a nucleophilic group such as a sulfhydryl or hydroxyl, that can be polarized and activated by the histidine residue of the catalytic triad [33]. Our alignment indicated that C91 of chUSP1 and flanking sequence are highly conserved in all USPs (Fig 2 and S4 Fig), and may provide the nucleophilic group, so C91 was replaced with both a non-nucleophilic alanine residue but also with a serine residue, which replaces the sulfydryl group with a more weakly nucleophilic hydroxyl group. Interestingly, while activity was completely abolished when C91 was replaced by a hydrophobic alanine residue, some activity was retained when this residue was replaced by a serine, suggesting that nucleophilicity at this position is required for chUSP1 activity, with a strong nucleophilic group being preferable. It was similar to hUSP1, where the mutation of C90A eliminated the deubiquitinating activity of hUSP1, but the mutant, hUSP1^C90S^ remains very low activity[31]. The reduction of activity from the mutation of C90S still impaired the cellular function of hUSP1[30]. The nucleophilic group of the cysteine located at the same site of other USPs have been certified to be indispensable for deubiquitinating activity through the mutation of cysteine to serine or alanine (S1 Table).

The alignment between chUSP1 and other USPs (S1 Table and S4 Fig) showed that two histidine residues, H594 and H603, are highly conserved, and correspond to H584 and H593 of hUSP1, respectively. The two histidine residues on these positions have been determined to be critical for catalytic activity of varied USPs (S1 Table)[31, 34–40]. The crystal structure of some USPs listed in S1 Table showed that both of these two histidine residues locate in the same region of catalytic core of USPs, and very close to catalytic cysteine residue or Gly76 of ubiquitin. This was the reason that these two histidine residues were chose as the candidates of catalytic histidine in current study. The mutation on H594 and H603 of chUSP1 impaired the deubiquitinating activity of the chUSP1/chUAF1 complex, while the interaction between chUSP1 and chUAF1 was not disrupted, suggesting that heterodimerization and catalytic activity are two distinct processes.

The mutation of H603 of chUSP1 did not completely erased deubiquitinating activity of chUSP1, where it was different from what was observed on the mutation of the corresponding site of hUSP1, H593Q, which fully eliminated the deubiquitinating activity of hUSP1[31]. However, the mutation on another candidate catalytic histidine, H594A, resulted in the loss of the deubiquitinating activity of chUSP1, although H584 of hUSP1, at the same position, has not been identified to be crucial for the deubiquitinating activity of hUSP1. The histidine residues at the same position of other USPs has been demonstrated to be essential to the deubiquitinating activity of these USPs, respectively[31, 34–40]. In addition to what we found, these reports may imply that these two histidine residues behaved differently for deubiquitinating activity on varied USPs, although they locate in highly conserved catalytic domain of chUSP1, hUPS1 and other USPs. It may also imply that chUSP1 functions differently from hUSP1. After all, they belong to different species and share varied amino acid sequence.

Generally, histidine residues in the catalytic core of an enzyme use the positive charge of their sidechain at neutral pH to promote enzyme-substrate interactions. Our results therefore suggest that H594 is more critical for such interactions in chUSP1 than H603, although these histidine residues are adjacent in the putative catalytic triad (S1 Table). The mutation of H603, a charged residue, to Alanine, a nonpolar residue, possibly altered the charge environment or the spatial architecture of catalytic core of chUSP1, subsequently impacted deubiquitinating activity of chUSP1.

The mutation D758A, in the putative catalytic core, reduced the DUB activity of the chUSP1/chUAF1 dimer but did not eliminate activity, suggesting that, while important, this residue is not indispensable for catalytic activity. This result is consistent with a study showing that a C-terminally truncated hUSP1, which lacks the region containing this residue, retained some deubiquitinating activity [13, 22]. Aspartic acid residues are often found in the active site of proteases, and their mechanism is thought to involve their carboxylic acid side chain providing a negative charge at neutral pH. It is therefore possible that the mutation of D758 to alanine, which has a neutral charge, reduces the binding between chUSP1/chUAF1 and substrate while not eliminating it completely.

Overall, our results suggest that C91, H594, and D758 comprise the catalytic triad of chUSP1, while C91 and H594 are essential for activity, D758 is less critical.

In addition, deubiquitinating activity also could be affected by reaction condition, such as NaCl concentration. To be comparable with the properties of hUSP1, the low NaCl concentration, 20 mM, was used in current study, which was the same as that used in very earlier studies on hUSP1[12, 13]. Recently, 100 mM of NaCl concentration was widely introduced in investigations related to hUSP1 or its inhibitors, although some studies related to kinetics or inhibition of DUBs including USP1, especially in inhibition studies, even did not use NaCl in HEPES buffer[18, 25]. However, Villamil et al. observed that the concentration of 50 mM to 1 M NaCl decreased *k*_cat_ values and increased *K*_M_ values of hUSP1[31]. This might be the reason of slight shift of *k*_cat_ and *K*_M_ values in different investigations on hUSP1[13, 31]. It has been reported that ionic strength affect Ub or Ub-like protein conformation or the interaction of substrate and protease. For example, SAMP1 and SAMP2, Ub-like proteins, could presents different conformation along with the change of ionic strength of solution[41, 42]. In absence of di-valent cations, such as copper or zinc, PrP^Sc^ were more likely to be digested by proteinase K in low NaCl concentration than in higher NaCl buffers, while high NaCl induces the conformation change and protease-resistant of PrP^Sc^ more easier than low NaCl[43]. K27 linkage di-Ub shows more resistance to deubiquitination than other linkage di-Ub because the unique conformation of K27 di-Ub confers low solvent accessibility[44]. The conformation change of Ub chain could modulate the recognition of activity of DUBs [1]. Thus, the alternation of Ub chain conformation by ionic strength may affect the substrate preference of USP. The effect of NaCl concentration on deubiquitinating reaction may need to be considered in future investigation on chUSP1.

Protein modification by Ub-chains with specific linkages leads to defined cellular outcomes. For instance, modification by a K48-linked Ub-chain usually targets the protein to the proteasome for degradation, modification by a K63-linked Ub-chain is involved in the regulation of DNA damage repair and genome stability, and modification of substrates of the anaphase-promoting complex by K11-linked Ub-chains helps to control progression through mitosis [45]. In the current study, we found that chUSP1/chUAF1 could efficiently hydrolyze K11-, K48- and K63-linked Ub-chains, suggesting that proteins with these specific Ub modifications may be targets for chUSP1/chUAF1 *in vivo*. Various linked type of Ub dimers were used for characterizing substrate preference of hUSP1ΔN *in vitro*[24]. This hUSP1ΔN showed similar substrate preference to chUSP1^FL^. However, hUSP1ΔN (75 nM) with or without hUAF1 could deconjugated K29 linked Ub dimer, while chUSP1^FL^ (1μM) or chUSP1^FL^ /chUAF1 complex (100 nM) showed no activity on K29-linked Ub dimers. chUSP1^FL^ /chUAF1 complex (100 nM) could completely hydrolyze K11-linked Ub dimer in reaction for 1 hr, but hUSP1ΔN /hUAF1 (75 nM) showed low activity for this Ub dimer in reaction lasted up to 2hr. This suggests that chUSP1 may deubiquitinate different subset of Ubs than its human counterpart. The reason probably is that USP1 protein encoded by two the species share unidentical amino acid sequence and different constitution of catalytic triad, just like what were observed in current study.

Conversely, chUSP1/chUAF1 did not efficiently hydrolyze other Ub-like substrates, and displayed a narrow target range. Therefore, we postulate that chUSP1/chUAF1 may regulate a specific subset of proteins involved in processes related to cellular proliferation, genomic DNA stability, protein degradation, and DNA damage repair within the cell. Such target specificity is advantageous in both drug development and in the development of investigative tools to examine the functions of various Ub modifications.

Binding of hUAF1 stabilizes hUSP1 and promotes interaction with PCNA-Ub and FANCD2-Ub [46], and the USP1/UAF1 complex promotes DNA double-strand break repair via homologous recombination [19]. Indeed, knocking out murine UAF1 causes a defect in homologous recombination and early embryonic lethality [47]. UAF1 and the USP1/UAF1 complex are also important in viral infection and pathogenesis, particularly in viral genomic integration and replication. The hUSP1/hUAF1 complex interacts with the human papilloma virus E1 DNA helicase to promote virus replication, and elimination of hUSP1/hUAF1 deubiquitinase activity dramatically impacted viral replication [48], and hUAF1 interacts with the *herpesvirus saimiri* Tip protein, promoting T cell receptor downregulation [14]. Previously, we identified chUAF1 and the putative chUSP1 protein in a screen of the preferred genomic integration sites of MDV, a herpesvirus with a tumorigenic role in chickens (unpublished observation). However, the substrate specificity of these enzymes, including their preferred Ub linkage, was unclear. We therefore aimed to characterize the interaction between chUSP1 and chUAF1, their substrate specificity, and enzymatic kinetics, in the hope of better understanding how their deubiquitinating activity is regulated during cellular proliferation, viral infection, and the maintenance of genomic stability *in vivo*.

Viral DUBs, like their cellular counterparts, regulate intracellular ubiquitination. Indeed, the N-terminal 1246 amino acids of BPLF1, the largest tegument encoded by the Epstein–Barr virus (3,149 amino acids in total), is highly conserved across all *Herpesviridae* and has deubiquitination activity. BPLF1 promotes Epstein-Barr virus infection and pathogenesis by mimicking USP1, deubiquitinating PCNA to disrupt the recruitment of DNA polymerases such as polη, and thus impeding DNA damage repair [49]. While there have been many advances in our understanding of the role of USP1 in the regulation of DNA damage repair and chromosome stability, including the development of an inhibitor, other aspects of USP1 function, including its participation in viral infection and pathogenesis, remain elusive. Viral genomic integration into the host cell genome is known to result in DNA damage and the activation of DNA repair pathways, and hUSP1 and hUAF1 are intimately involved in DNA damage repair and genomic stability, as well as in carcinoma progression in some cells. It will therefore be interesting, in the future, to investigate whether chUSP1 and chUAF1 are able to modulate viral genomic integration in chickens, particularly where viral infection is known to be carcinogenic.

In summary, the *in vitro* characterization of chUSP1 presented here may provide a reference with which to understand the *in vivo* deubiquitination activity of USP1, and may help to further our understanding of the mechanisms underpinning viral infection, including genomic integration, tumorigenesis, and immunosuppression.

## Supporting information

S1 Fig Western blot confirming the identity of the intracellularly co-expressed chUSP1 and chUAF1 proteins observed in panels A-D of Fig 4. Upper panel: the indicated chUSP1 proteins were detected in complexes using an anti-hexa-His tag primary antibody. Lower panel: chUAF1 protein present in complexes was detected using an anti-chUAF1 primary antibody. Uninfected Sf9 cells were used as controls.
(TIF)

S2 Fig The Michaelis–Menten plots and Lineweaver-Burk plots of chUSP1 or chUSP1/chUAF1 complexes. (A), chUSP1^FL^ alone. (B), chUSP1^FL^/chUAF1. (C), chUSP1^C91S^/chUAF1. (D), chUSP1^H603A^/chUAF1. (E), chUSP1^D758A^/chUAF1.
(TIF)

S3 Fig The chUSP1FL catalytic core mutants showing no activity. Fluorescence intensity traces showing the release of AMC from Ub-AMC over time with chUSP1^C91A^ /chUAF1(gray), chUSP1^H594A^ /chUAF1(red), chUSP1^CH91,594SA^ /chUAF1(blue) protein complexes, and chUSP1FL/chUAF1 with the inhibitor Ub-VS(green).
(TIF)

S4 Fig The alignment of catalytic domain in USPs. The multiple alignment of catalytic domain between chUSP1 and other USPs by CLUSTAL OMEGA (http://www.ebi.ac.uk/Tools/msa/clustalo/), the gaps or otherwise nonconserved sequences were omitted. The numbers on the top of boxes indicate the position of mutated catalytic residues in chUSP1 (C91, H594, H603 and D758). The conserved putative catalytic residues were highlighted in different color, Cys in red, His corresponding to His594 in chicken in blue, His corresponding to H603 in yellow, and Asp corresponding to D758 in green.
(TIF)

S1 Table The reported sites of catalytic residues of USPs. The USPs on which the catalytic residues were confirmed by investigations were listed in S1 Table. Corresponding to S4 Fig, these putative catalytic residues were highlighted in different color, Cys in red, His corresponding to His594 in chicken in blue, His corresponding to H603 in yellow.
(DOC)
