# Supplementary figures and images for: Characterization of the deubiquitination activity and substrate specificity of the chicken ubiquitin-specific protease 1/USP associated factor 1 complex
Source: PLoS One. 2017 Nov 1;12(11):e0186535. doi: 10.1371/journal.pone.0186535 (PMC5665528; doi:10.1371/journal.pone.0186535)

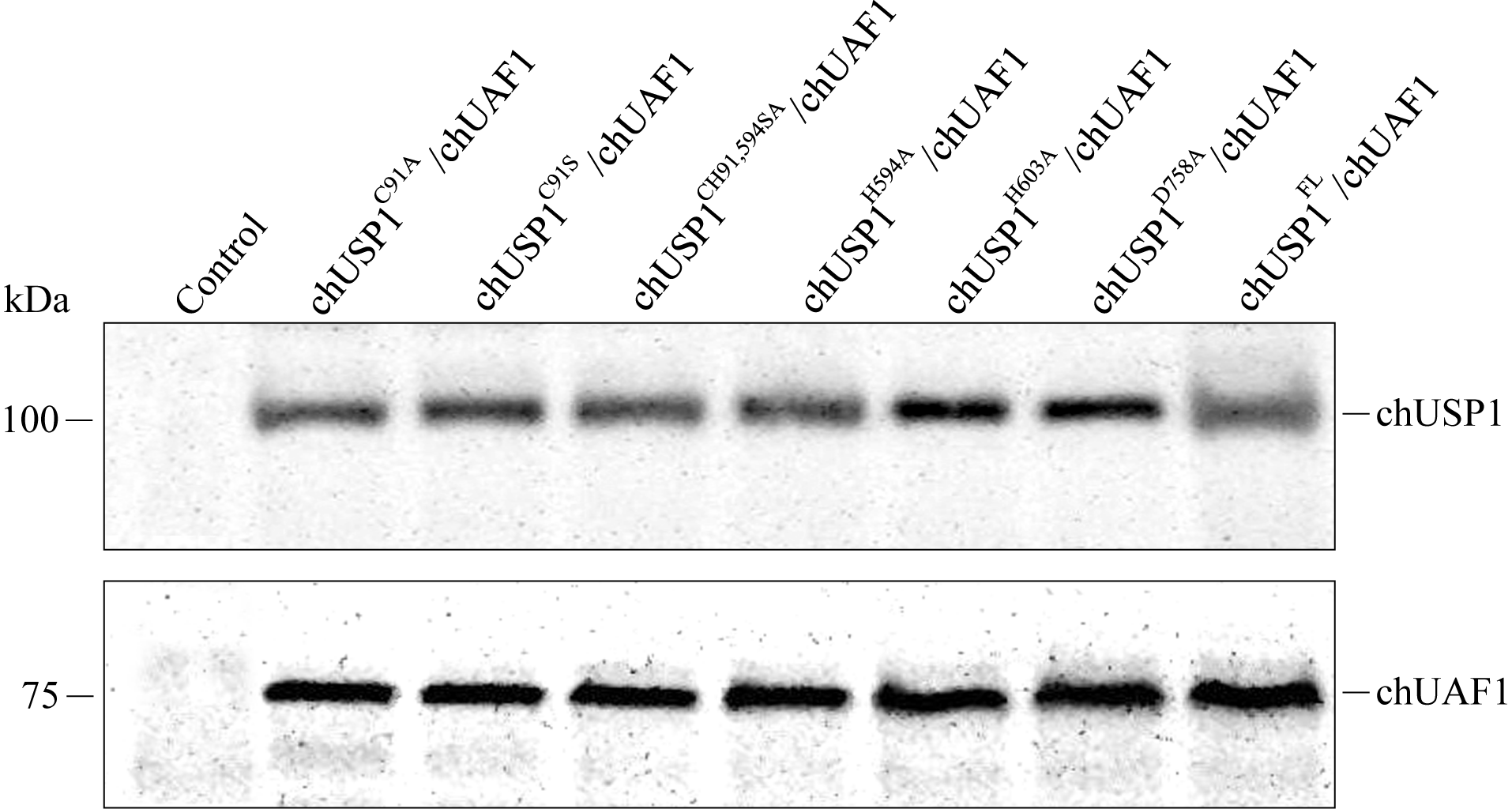

Supplement: S1 Fig — Upper panel: the indicated chUSP1 proteins were detected in complexes using an anti-hexa-His tag primary antibody. Lower panel: chUAF1 protein present in complexes was detected using an anti-chUAF1 primary antibody. Uninfected Sf9 cells were used as controls. (TIF) [file pone.0186535.s001.tif]

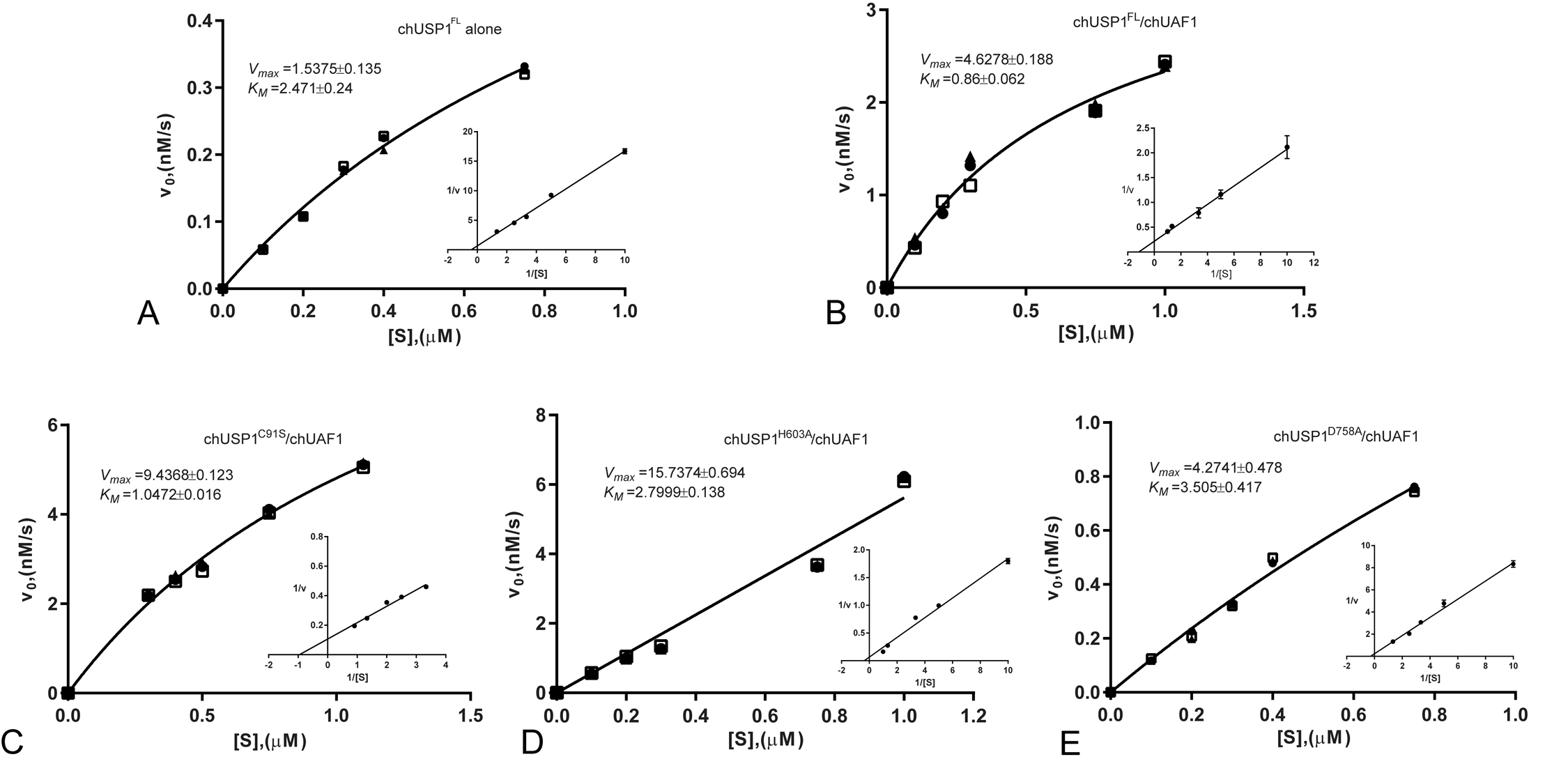

Supplement: S2 Fig — (A), chUSP1FL alone. (B), chUSP1FL/chUAF1. (C), chUSP1C91S/chUAF1. (D), chUSP1H603A/chUAF1. (E), chUSP1D758A/chUAF1. (TIF) [file pone.0186535.s002.tif]

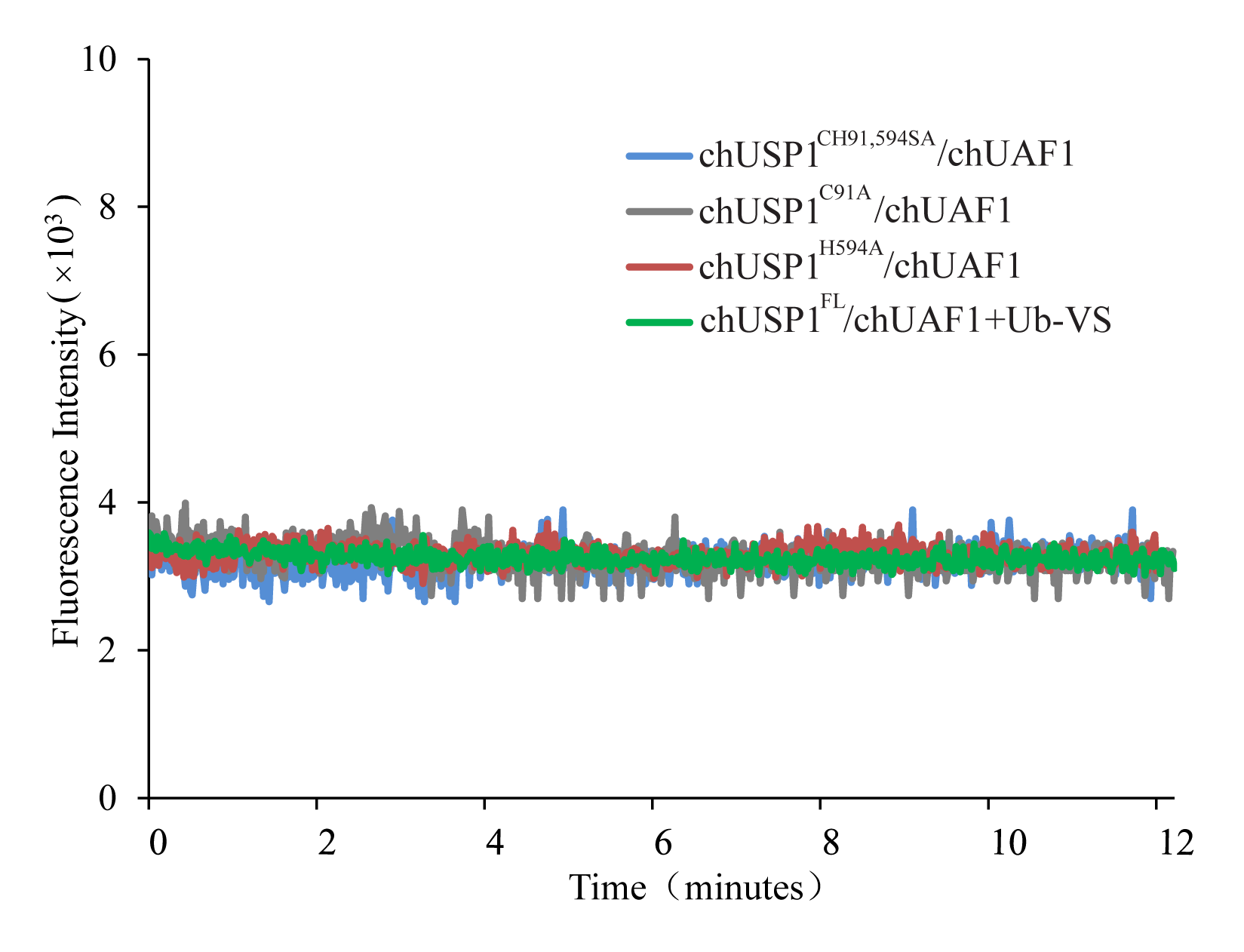

Supplement: S3 Fig — Fluorescence intensity traces showing the release of AMC from Ub-AMC over time with chUSP1C91A /chUAF1(gray), chUSP1H594A /chUAF1(red), chUSP1CH91,594SA /chUAF1(blue) protein complexes, and chUSP1FL/chUAF1 with the inhibitor Ub-VS(green). (TIF) [file pone.0186535.s003.tif]

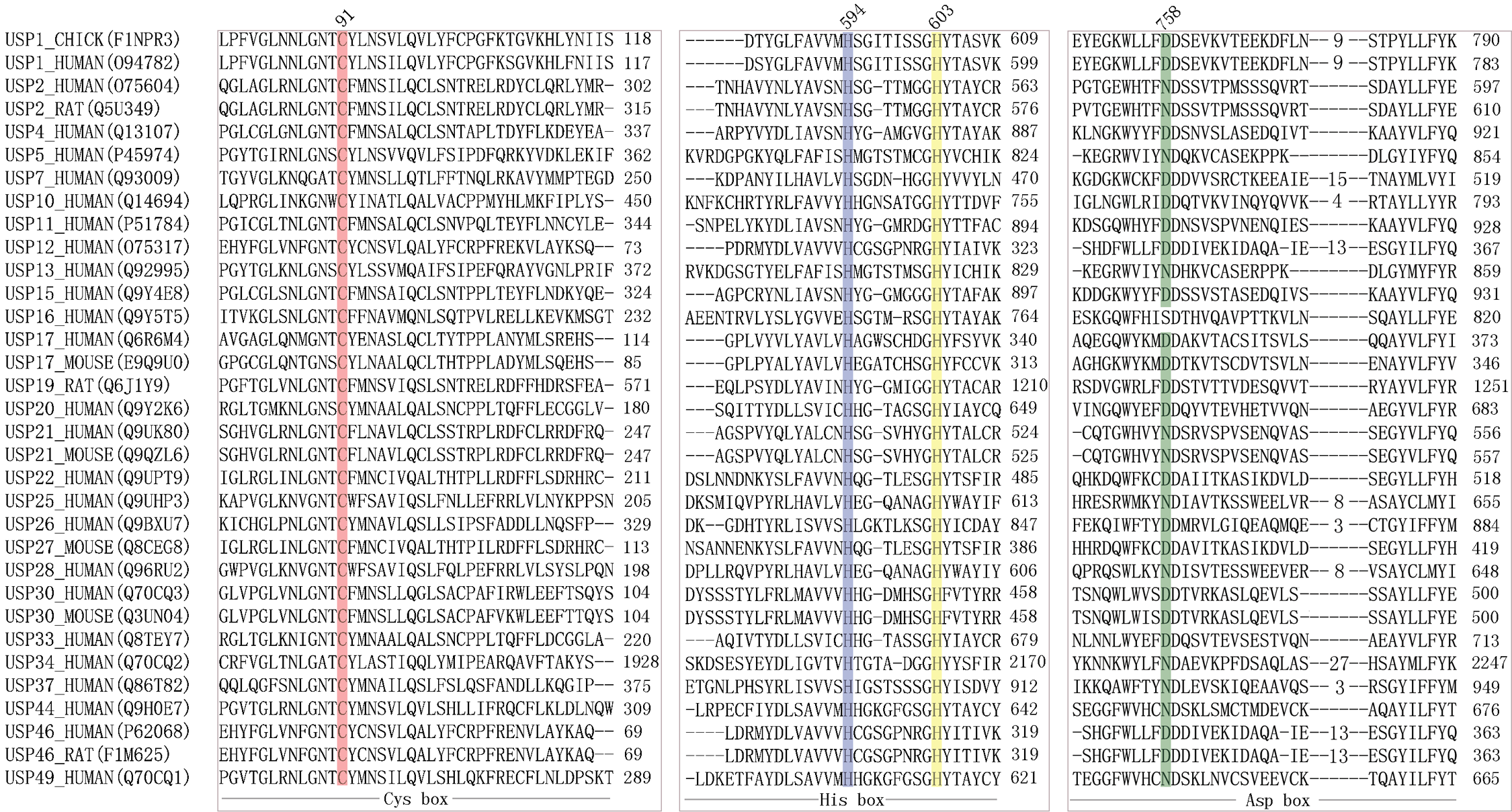

Supplement: S4 Fig — The multiple alignment of catalytic domain between chUSP1 and other USPs by CLUSTAL OMEGA (http://www.ebi.ac.uk/Tools/msa/clustalo/), the gaps or otherwise nonconserved sequences were omitted. The numbers on the top of boxes indicate the position of mutated catalytic residues in chUSP1 (C91, H594, H603 and D758). The conserved putative catalytic residues were highlighted in different color, Cys in red, His corresponding to His594 in chicken in blue, His corresponding to H603 in yellow, and Asp corresponding to D758 in green. (TIF) [file pone.0186535.s004.tif]
