# Supplementary material for: Characterization of the deubiquitination activity and substrate specificity of the chicken ubiquitin-specific protease 1/USP associated factor 1 complex
Source: PLoS One. 2017 Nov 1;12(11):e0186535. doi: 10.1371/journal.pone.0186535 (PMC5665528; doi:10.1371/journal.pone.0186535)
Supplement: S1 Table — The USPs on which the catalytic residues were confirmed by investigations were listed in S1 Table. Corresponding to S4 Fig, these putative catalytic residues were highlighted in different color, Cys in red, His corresponding to His594 in chicken in blue, His corresponding to H603 in yellow. (DOC) [file pone.0186535.s005.doc]

**S1 Fig. Western blot confirming the identity of the intracellularly co-expressed chUSP1 and chUAF1 proteins observed in panels A-D of Fig 4.** Upper panel: the indicated chUSP1 proteins were detected in complexes using an anti-hexa-His tag primary antibody. Lower panel: chUAF1 protein present in complexes was detected using an anti-chUAF1 primary antibody. Uninfected Sf9 cells were used as controls.

**S2 Fig. The Michaelis–Menten plots and Lineweaver-Burk plots of chUSP1 or chUSP1/chUAF1 complexes.** (A), chUSP1^FL^ alone. (B), chUSP1^FL^/chUAF1. (C), chUSP1^C91S^/chUAF1. (D), chUSP1^H603A^/chUAF1. (E), chUSP1^D758A^/chUAF1.

**S3 Fig. The chUSP1FL catalytic core mutants showing no activity.** Fluorescence intensity traces showing the release of AMC from Ub-AMC over time with chUSP1^C91A^ /chUAF1(gray), chUSP1^H594A^ /chUAF1(red), chUSP1^CH91,594SA^ /chUAF1(blue) protein complexes, and chUSP1FL/chUAF1 with the inhibitor Ub-VS(green).

**S4 Fig. The alignment of catalytic domain in USPs.** The multiple alignment of catalytic domain between chUSP1 and other USPs by CLUSTAL OMEGA (http://www.ebi.ac.uk/Tools/msa/clustalo/), the gaps or otherwise noncon- served sequences were omitted. The numbers on the top of boxes indicate the position of mutated catalytic residues in chUSP1 (C91, H594, H603 and D758). The conserved putative catalytic residues were highlighted in different color, Cys in red, His corresponding to His594 in chicken in blue, His corresponding to H603 in yellow, and Asp corresponding to D758 in green.

**S1 Table. The reported sites of catalytic residues of USPs**

| DUBs | UniProtKB Entry# | PDB # | Cys | His | His |
| --- | --- | --- | --- | --- | --- |
| chUSP1 | F1NPR3 |  | C91A | H594A | H603A |
|  |  |  | C91S |  |  |
| hUSP1 | O94782 |  | C90A[1] |  | H593Q[1] |
|  |  |  | C90S[1-3] |  |  |
| hUSP2 | O75604 | 3NHE, 2HD5 | C276A[4] | H549A[5, 6] |  |
| rat USP2(UBP69) | Q5U349 |  | C289S[7] |  |  |
| hUSP4 | Q13107 | 3JYU, 2Y6E | C311A[8, 9] |  |  |
| hUSP5 | P45974 | 3IHP | C335A[10] |  |  |
| hUSP7 | Q93009 | 5FWI | C223A[11] | H456A[11] | H464A[11] |
| hUSP10 | Q14694 |  | C424A[12] |  |  |
| hUSP11 | P51784 |  | C275S[13] |  |  |
| hUSP12 | O75317 | 5L8W | C48A[14] |  |  |
|  |  |  | C48S[15] |  |  |
| hUSP13 | Q92995 |  | C345A[16] | H814A[16] | H823A[16] |
| hUSP15 | Q9Y4E8 |  | C269A[17] |  |  |
|  |  |  | C269S[17] |  |  |
| hUSP16 | Q9Y5T5 |  | C205A[18] |  |  |
| hUSP17 | Q6R6M4 |  | C89S[19] |  |  |
| mUSP17 | E9Q9U0 |  | C60S[20] |  |  |
| ratUSP19 | Q6J1Y9 |  | C545A[21] |  |  |
| hUSP20 | Q9Y2K6 |  | C154S[22] |  | H643Q[22] |
| hUSP21 | Q9UK80 | 3I3T, 2Y5B, 3MTN | C221A[23] |  |  |
| mUSP21 | Q9QZL6 |  | C221A[24] |  | H519A[24] |
| hUSP25 | Q9UHP3 |  | C178S[25] |  |  |
| hUSP26 | Q9BXU7 |  | C304S[26] |  |  |
| mUSP27 | Q8CEG8 |  | C87A[27] |  |  |
| hUSP28 | Q96RU2 |  | C171A[28] |  |  |
| hUSP30 | Q70CQ3 |  | C77S[29] |  |  |
| mUSP30 | Q3UN04 |  | C77S[30] | H452A[30] |  |
| hUSP33 | Q8TEY7 |  | C194S[22] |  | H673Q[22] |
| hUSP34 | Q70CQ2 |  | C1903S[31] |  |  |
| hUSP37 | Q86T82 |  | C350S[32] |  |  |
| hUSP44 | Q9H0E7 |  | C282A[33] |  |  |
|  |  |  | C282S[34] |  |  |
| rat UBP45(UBP2) | Q5U349-2 |  | C67S[7] |  |  |
| hUSP46 | P62068 | 5L8H, 5CVM, 5CVO | C44S[35, 36] |  |  |
| ratUSP46 | F1M625 |  | C44S[35, 36] |  |  |
| hUSP49 | Q70CQ1 |  | C262A[37] |  |  |

The USPs on which the catalytic residues were confirmed by investigations were listed in S1 Table. Corresponding to S4 Fig., these putative catalytic residues were highlighted in different color, Cys in red, His corresponding to His594 in chicken in blue, His corresponding to H603 in yellow.

**References for S1 Table**

1. Villamil MA, Chen J, Liang Q, Zhuang Z. A noncanonical cysteine protease USP1 is activated through active site modulation by USP1-associated factor 1. Biochemistry. 2012; 51: 2829-2839.

2. Olazabal-Herrero A, Garcia-Santisteban I, Rodriguez JA. Structure-function analysis of USP1: insights into the role of Ser313 phosphorylation site and the effect of cancer-associated mutations on autocleavage. Mol Cancer. 2015; 14: 33.

3. Jung JK, Jang SW, Kim JM. A novel role for the deubiquitinase USP1 in the control of centrosome duplication. Cell Cycle. 2016; 15: 584-592.

4. Shan J, Zhao W, Gu W. Suppression of cancer cell growth by promoting cyclin D1 degradation. Mol Cell. 2009; 36: 469-476.

5. Stevenson LF, Sparks A, Allende‐Vega N, Xirodimas DP, Lane DP, Saville MK. The deubiquitinating enzyme USP2a regulates the p53 pathway by targeting Mdm2. The EMBO Journal. 2007; 26: 976.

6. Allende-Vega N, Sparks A, Lane DP, Saville MK. MdmX is a substrate for the deubiquitinating enzyme USP2a. Oncogene. 2009; 29: 432-441.

7. Park KC, Kim JH, Choi EJ, Min SW, Rhee S, Baek SH, et al. Antagonistic regulation of myogenesis by two deubiquitinating enzymes, UBP45 and UBP69. Proc Natl Acad Sci U S A. 2002; 99: 9733-9738.

8. Wada K, Tanji K, Kamitani T. Oncogenic protein UnpEL/Usp4 deubiquitinates Ro52 by its isopeptidase activity. Biochem Biophys Res Commun. 2006; 339: 731-736.

9. Wada K, Kamitani T. UnpEL/Usp4 is ubiquitinated by Ro52 and deubiquitinated by itself. Biochemical and Biophysical Research Communications. 2006; 342: 253-258.

10. Reyes-Turcu FE, Shanks JR, Komander D, Wilkinson KD. Recognition of polyubiquitin isoforms by the multiple ubiquitin binding modules of isopeptidase T. J Biol Chem. 2008; 283: 19581-19592.

11. Hu M, Li P, Li M, Li W, Yao T, Wu J-W, et al. Crystal Structure of a UBP-Family Deubiquitinating Enzyme in Isolation and in Complex with Ubiquitin Aldehyde. Cell. 2002; 111: 1041-1054.

12. Bomberger JM, Barnaby RL, Stanton BA. The deubiquitinating enzyme USP10 regulates the post-endocytic sorting of cystic fibrosis transmembrane conductance regulator in airway epithelial cells. J Biol Chem. 2009; 284: 18778-18789.

13. Schoenfeld AR, Apgar S, Dolios G, Wang R, Aaronson SA. BRCA2 Is Ubiquitinated In Vivo and Interacts with USP11, a Deubiquitinating Enzyme That Exhibits Prosurvival Function in the Cellular Response to DNA Damage. Molecular and Cellular Biology. 2004; 24: 7444-7455.

14. Li H, Lim KS, Kim H, Hinds TR, Jo U, Mao H, et al. Allosteric Activation of Ubiquitin-Specific Proteases by beta-Propeller Proteins UAF1 and WDR20. Mol Cell. 2016; 63: 249-260.

15. Cohn MA, Kee Y, Haas W, Gygi SP, D'Andrea AD. UAF1 is a subunit of multiple deubiquitinating enzyme complexes. J Biol Chem. 2009; 284: 5343-5351.

16. Scortegagna M, Subtil T, Qi J, Kim H, Zhao W, Gu W, et al. USP13 enzyme regulates Siah2 ligase stability and activity via noncatalytic ubiquitin-binding domains. J Biol Chem. 2011; 286: 27333-27341.

17. Inui M, Manfrin A, Mamidi A, Martello G, Morsut L, Soligo S, et al. USP15 is a deubiquitylating enzyme for receptor-activated SMADs. Nat Cell Biol. 2011; 13: 1368-1375.

18. Cai SY, Babbitt RW, Marchesi VT. A mutant deubiquitinating enzyme (Ubp-M) associates with mitotic chromosomes and blocks cell division. Proc Natl Acad Sci U S A. 1999; 96: 2828-2833.

19. Burrows JF, McGrattan MJ, Rascle A, Humbert M, Baek KH, Johnston JA. DUB-3, a cytokine-inducible deubiquitinating enzyme that blocks proliferation. J Biol Chem. 2004; 279: 13993-14000.

20. Baek KH, Kim MS, Kim YS, Shin JM, Choi HK. DUB-1A, a novel deubiquitinating enzyme subfamily member, is polyubiquitinated and cytokine-inducible in B-lymphocytes. J Biol Chem. 2004; 279: 2368-2376.

21. Lu Y, Adegoke OA, Nepveu A, Nakayama KI, Bedard N, Cheng D, et al. USP19 deubiquitinating enzyme supports cell proliferation by stabilizing KPC1, a ubiquitin ligase for p27Kip1. Mol Cell Biol. 2009; 29: 547-558.

22. Berthouze M, Venkataramanan V, Li Y, Shenoy SK. The deubiquitinases USP33 and USP20 coordinate beta2 adrenergic receptor recycling and resensitization. EMBO J. 2009; 28: 1684-1696.

23. Gong L, Kamitani T, Millas S, Yeh ET. Identification of a novel isopeptidase with dual specificity for ubiquitin- and NEDD8-conjugated proteins. J Biol Chem. 2000; 275: 14212-14216.

24. Nakagawa T, Kajitani T, Togo S, Masuko N, Ohdan H, Hishikawa Y, et al. Deubiquitylation of histone H2A activates transcriptional initiation via trans-histone cross-talk with H3K4 di- and trimethylation. Genes Dev. 2008; 22: 37-49.

25. Denuc A, Bosch-Comas A, Gonzalez-Duarte R, Marfany G. The UBA-UIM domains of the USP25 regulate the enzyme ubiquitination state and modulate substrate recognition. PLoS One. 2009; 4: e5571.

26. Dirac AM, Bernards R. The deubiquitinating enzyme USP26 is a regulator of androgen receptor signaling. Mol Cancer Res. 2010; 8: 844-854.

27. Weber A, Heinlein M, Dengjel J, Alber C, Singh PK, Hacker G. The deubiquitinase Usp27x stabilizes the BH3-only protein Bim and enhances apoptosis. EMBO Rep. 2016; 17: 724-738.

28. Zhang D, Zaugg K, Mak TW, Elledge SJ. A role for the deubiquitinating enzyme USP28 in control of the DNA-damage response. Cell. 2006; 126: 529-542.

29. Nakamura N, Hirose S. Regulation of mitochondrial morphology by USP30, a deubiquitinating enzyme present in the mitochondrial outer membrane. Mol Biol Cell. 2008; 19: 1903-1911.

30. Yue W, Chen Z, Liu H, Yan C, Chen M, Feng D, et al. A small natural molecule promotes mitochondrial fusion through inhibition of the deubiquitinase USP30. Cell Res. 2014; 24: 482-496.

31. Lui TT, Lacroix C, Ahmed SM, Goldenberg SJ, Leach CA, Daulat AM, et al. The ubiquitin-specific protease USP34 regulates axin stability and Wnt/beta-catenin signaling. Mol Cell Biol. 2011; 31: 2053-2065.

32. Huang X, Summers Matthew K, Pham V, Lill Jennie R, Liu J, Lee G, et al. Deubiquitinase USP37 Is Activated by CDK2 to Antagonize APCCDH1 and Promote S Phase Entry. Molecular Cell. 2011; 42: 511-523.

33. Stegmeier F, Rape M, Draviam VM, Nalepa G, Sowa ME, Ang XL, et al. Anaphase initiation is regulated by antagonistic ubiquitination and deubiquitination activities. Nature. 2007; 446: 876-881.

34. Suresh B, Ramakrishna S, Lee HJ, Choi JH, Kim JY, Ahn WS, et al. K48- and K63-linked polyubiquitination of deubiquitinating enzyme USP44. Cell Biol Int. 2010; 34: 799-808.

35. Zhang W, Tian QB, Li QK, Wang JM, Wang CN, Liu T, et al. Lysine 92 amino acid residue of USP46, a gene associated with 'behavioral despair' in mice, influences the deubiquitinating enzyme activity. PLoS One. 2011; 6: e26297.

36. Yin J, Schoeffler Allyn J, Wickliffe K, Newton K, Starovasnik Melissa A, Dueber Erin C, et al. Structural Insights into WD-Repeat 48 Activation of Ubiquitin-Specific Protease 46. Structure. 2015; 23: 2043-2054.

37. Zhang Z, Jones A, Joo HY, Zhou D, Cao Y, Chen S, et al. USP49 deubiquitinates histone H2B and regulates cotranscriptional pre-mRNA splicing. Genes Dev. 2013; 27: 1581-1595.
